# Gender-affirming hormone therapy induces specific DNA methylation changes in blood

**DOI:** 10.1186/s13148-022-01236-4

**Published:** 2022-02-17

**Authors:** Rebecca Shepherd, Ingrid Bretherton, Ken Pang, Toby Mansell, Anna Czajko, Bowon Kim, Amanda Vlahos, Jeffrey D. Zajac, Richard Saffery, Ada Cheung, Boris Novakovic

**Affiliations:** 1 Molecular Immunity, Infection and Immunity Theme, Murdoch Children’s Research Institute, Royal Children’s Hospital, Parkville, VIC Australia; 2 Department of Medicine (Austin Health), The University of Melbourne, Parkville, VIC Australia; 3 Department of Endocrinology, Austin Health, Heidelberg, VIC Australia; 4 Brain and Mitochondrial Research, Murdoch Children’s Research Institute, Royal Children’s Hospital, Parkville, VIC Australia; 5 Inflammation Division, The Walter and Eliza Hall Institute of Medical Research, Parkville, VIC Australia; 6 Department of Adolescent Medicine, Royal Children’s Hospital, Parkville, VIC Australia; 7 Department of Paediatrics, The University of Melbourne, Parkville, VIC Australia

**Keywords:** DNA methylation, EPIC array, Transgender, Masculinization, Feminization, GAHT, Epigenetics, Gender, Immunity

## Abstract

**Background:**

DNA methylation is an epigenetic mark that is influenced by underlying genetic profile, environment, and ageing. In addition to X-linked DNA methylation, sex-specific methylation patterns are widespread across autosomal chromosomes and can be present from birth or arise over time. In individuals where gender identity and sex assigned at birth are markedly incongruent, as in the case of transgender people, feminization or masculinization may be sought through gender-affirming hormone therapy (GAHT). GAHT is a cornerstone of transgender care, yet no studies to date have investigated its effect on genome-wide methylation. We profiled genome-wide DNA methylation in blood of transgender women (*n* = 13) and transgender men (*n* = 13) before and during GAHT (6 months and 12 months into feminizing or masculinizing hormone therapy).

**Results:**

We identified several thousand differentially methylated CpG sites (DMPs) (Δ*β* ≥ 0.02, unadjusted *p* value < 0.05) and several differentially methylated regions (DMRs) in both people undergoing feminizing and masculinizing GAHT, the vast majority of which were progressive changes over time. X chromosome and sex-specific autosomal DNA methylation patterns established in early development are largely refractory to change in association with GAHT, with only 3% affected (Δ*β* ≥ 0.02, unadjusted *p* value < 0.05). The small number of sex-specific DMPs that were affected by GAHT were those that become sex-specific during the lifetime, known as sex-and-age DMPs, including DMRs in *PRR4* and *VMP1* genes. The GAHT-induced changes at these sex-associated probes consistently demonstrated a shift towards the methylation signature of the GAHT-naïve opposite sex, and we observed enrichment of previously reported adolescence-associated methylation changes.

**Conclusion:**

We provide evidence for GAHT inducing a unique blood methylation signature in transgender people. This study advances our understanding of the complex interplay between sex hormones, sex chromosomes, and DNA methylation in the context of immunity. We highlight the need to broaden the field of ‘sex-specific’ immunity beyond cisgender males and cisgender females, as transgender people on GAHT exhibit a unique molecular profile.

**Supplementary Information:**

The online version contains supplementary material available at 10.1186/s13148-022-01236-4.

## Background

In the era of personalized medicine, inclusivity of all genders and sexes in research is vital for providing equitable and inclusive health care [1]. Although often used interchangeably, gender and sex are separate constructs. ‘Gender identity’ refers to one’s self-perception of gender, while ‘sex’ refers to the underpinning biological features, which is assigned at birth and defined by reproductive anatomy or sex chromosomes [2, 3]. Gender dysphoria occurs when an individual’s gender identity and sex assigned at birth do not align. In those where these are markedly and consistently incongruent, as in the case of transgender individuals, feminization or masculinization may be sought through gender-affirming hormone therapy (GAHT). In those seeking feminization, GAHT includes oestrogen (in the presence of absence of progesterone) in combination with anti-androgens, while testosterone therapy is used for those seeking masculinization.

A wealth of research exists on ‘sex-specific’ immunity, including evidence of sex-specific vaccine and infection responses (reviewed in [4, 5]). Additionally, sexual dimorphism exists within several immunological diseases, such as the heightened prevalence of some autoimmune and inflammatory diseases among adult individuals presumed female at birth compared to adult individuals presumed male at birth [6]. However, these findings are based on binary comparisons based on sex, and it is important to broaden the current understanding of immunity to include transgender individuals. Moreover, sex hormones have an immunomodulatory role (reviewed in [7–9]), and therefore the effects of GAHT warrant investigation as it marks a period of profound change in the internal hormonal milieu.

DNA methylation is an epigenetic mark that influences gene expression in a context-specific manner [10]. This mark is highly dynamic during pre-implantation development and during cell differentiation [11], but is also susceptible to environmental factors throughout the lifetime [12]. The inactivation of the second X chromosome in females is an example of a co-ordinated sex-specific epigenetic remodelling process. This process involves a non-coding RNA, chromatin remodelling, and finally the addition of DNA methylation on the inactive X [13]. In addition to X inactivation-associated DNA methylation differences [13, 14], sex-specific (XX females versus XY males) DNA methylation patterns on autosomal chromosomes have been reported in a range of cell types [15–18]. At least some of these are potentially due to hormonal change, but these are hard to delineate from genetic influence, as hormones are regulated by biological sex in isolation. Periods of hormonal change have been shown to induce changes in DNA methylation over time, including pregnancy [19], puberty [20, 21], menopause, and menopausal hormone therapy [22, 23]. Analysing GAHT-induced DNA methylation change provides a unique model to study the effect of hormones separate from genetics. Here we report the first Epigenome-wide association study (EWAS) of GAHT, and describe the longitudinal changes observed in the blood methylome of 13 transgender men and 13 transgender women newly commencing GAHT at baseline, 6 months, and 12 months.

## Results

### Gender-affirming hormone therapy induces progressive changes in blood DNA methylation

**Fig. 1:**
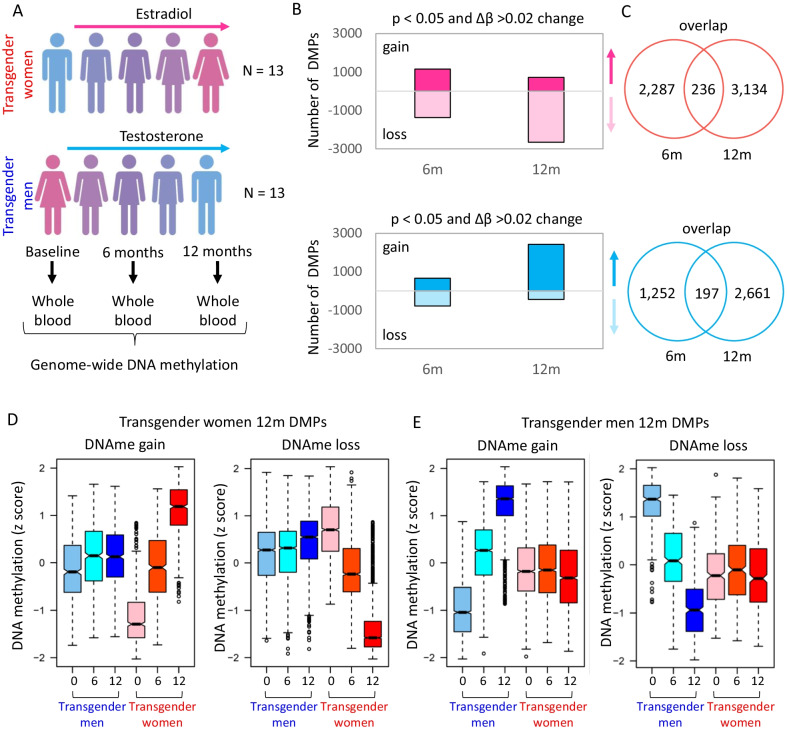
Gender-affirming hormone therapy model induced progressive change in blood DNA methylation. **A** Longitudinal GAHT model with blood collection at baseline (before hormone therapy), and 6 and 12 months after treatment. In total, samples from 13 transgender women and 13 transgender men were included in the study. **B** Number of differentially methylated probes (DMPs) with an unadjusted *p* value cut-off < 0.05 and a Δ*β* cut-off of ≥ 0.02. **C** Venn diagram showing the overlap between 6 and 12 m DMPs. **D** Boxplots of DNA methylation z-scores at DMPs that gain or lose DNA methylation at 12 months compared to baseline in transgender women. At 6 months, these probes show an intermediate level of DNA methylation. **E** Boxplots of DNA methylation z-scores at DMPs that gain or lose DNA methylation in transgender men

To investigate whether DNA methylation levels in blood change in response to GAHT, we analysed epigenome-wide methylation data in transgender women commencing feminizing hormone therapy and transgender men commencing masculinizing hormone therapy (Fig. 1A). In both models, we profiled longitudinal samples of 13 individuals at baseline, and after 6 months (6 m) and 12 months (12 m) GAHT (Table 1, Additional file 10: Fig. S1). No probes reached significance after adjustment for multiple testing in both feminizing and masculinizing GAHT analysis.

**Table 1:** Age, body mass index and baseline, 6- and 12-month biochemistry in transgender women and transgender men

	Transgender Women (n=13) (TW)	Transgender Men (n=13) (TM)
Age (years)	29.0 (22.0, 61.0)	23.0 (21.0, 24.0)
BMI (kg/m²)	24.2 (22.3, 24.6)	22.8 (21.8, 30.5)

	Baseline (TW)	6 months﻿ (TW)	12 months﻿ (TW)	Baseline (TM)	6 months﻿ (TM)	12 months﻿ (TM)
**Biochemistry**						
FSH (IU/L)	5.2 (2.6, 7.4)	0.8 (0.1, 1.2)	0.2 (0.1, 0.8)	6.4 (3.3, 7.2)	5.4 (4.9, 6.3)	4.1 (3.5, 5.4)
LH (IU/L)	6.1 (5.4, 6.7)	2.0 (0.1, 3.9)	0.1 (0.1, 2.2)	7.5 (5.9, 8.3)	7.2 (6.2, 10.0)	4.7 (2.1, 7.0)
E2 (pmol/L)	56.0 (50.0, 76.0)	259.0 (196.0,393.0)	340.0 (294.0, 419.0)	210.0 (90.5, 460.5)	165.0 (92.0, 171.0)	157.0 (120.0, 197.0)
T (nmol/L)	19.6 (19.8, 20.4)	0.7 (0.4, 1.6)	0.5 (0.4, 0.7)	1.6 (1.2, 1.7)	20.9 (15.3, 27.1)	20.4 (17.5, 29.2)
SHBG (nmol/L)	51.0 (43.5, 54.0)	74.0 (49.0, 105.0)	76.0 (53.0, 98.0)	51.0 (39.5, 74.5)	37.0 (21.0, 56.0)	33.0 (23.0, 62.0)

Expressed as median (interquartile range). Follicle-stimulating hormone (FSH), luteinizing hormone (LH), estradiol (E2), testosterone (T), sex hormone binding globulin (SHBG)

In transgender women, we identified 5657 differentially methylated probes (DMPs) after 6 m or 12 m of feminizing GAHT (*p* value (unadjusted) < 0.05 and mean Δ*β* ≥ 0.02) (Fig. 1B, Additional file 1: Table S1). In transgender men, we identified 4110 DMPs (*p* value (unadjusted) < 0.05 and mean Δ*β* > 0.02) (Fig. 1B, Additional file 2: Table S2). The mean methylation difference for the top DMPs in transgender women and transgender men and the associated closest genes, are shown in Additional file 10: Fig. S2A and S2B. There was little overlap between 6 and 12 m DMPs (Fig. 1C, Additional file 1: Table S1), however when DNA methylation levels for the 12 m DMPs were plotted, the 6 m time point showed an intermediate level of methylation (red plots in Fig. 1D, and blue plots in Fig. 1E), indicating progressive change over time. There was a strong correlation in Δβ between ‘baseline v 6 months’ and ‘baseline v 12 months’, with no anti-correlating DMPs (Additional file 10: Fig. S3A and S3B, and Additional file 2: Table S2), which is also evident in a direction-of-change stratified Venn diagram (Additional file 10: Fig. S3C and S3D). Based on these DMPs the baseline group separates from the 6-month and 12-month groups, which cluster together in both transgender women and transgender men by principal component analysis (Additional file 10: Fig. S3E and S3F). In summary, DNA methylation profile is remodelled by 6 months post-GAHT in a hormone-specific manner, and this pattern is maintained or enhanced by 12 months.

### Temporal GAHT-associated DNA methylation dynamics

While the primary pattern of change following GAHT was a progressive gain or loss of DNA methylation, due to the poor overlap between significant DMPs (Fig. 1C) we wanted to explore temporal DNA methylation dynamics (Additional file 10: Fig. S4). Using k-means clustering, we identified 8 unique DMP clusters in feminizing GAHT (Additional file 10: Fig. S4A) and 6 unique DMP clusters in masculinizing GAHT (Additional file 10: Fig. S4B). The largest cluster for feminizing GAHT was progressive loss of DNA methylation (1193 DMPs), while the largest cluster for masculinizing GAHT was progressive gain of DNA methylation (1154 DMPs). We additionally identified transient clusters (gain in DNA methylation at 6 m, followed by return to baseline levels at 12 m), as well as those that show early change in DNA methylation at 6 m and maintain it to 12 m. We annotated the DMPs in Additional file 1: Table S1 and Additional file 2: Table S2 with the cluster they belong to (Additional file 1: Table S1 and Additional file 2: Table S2) and performed gene ontology analysis using genes near these clusters (Additional file 3: Table S3). In addition to this approach, we also used a time-course model in limma to identify DMPs that were significant for a linear change across all three time points (Additional file 4: Table S4 and Additional file 5: Table S5). Time could also have been modelled as a factor to derive results similar to the stratified analysis, but this was not performed in this study’.

### Feminizing and masculinizing GAHT remodel DNA methylation profiles in opposing directions

**Fig. 2:**
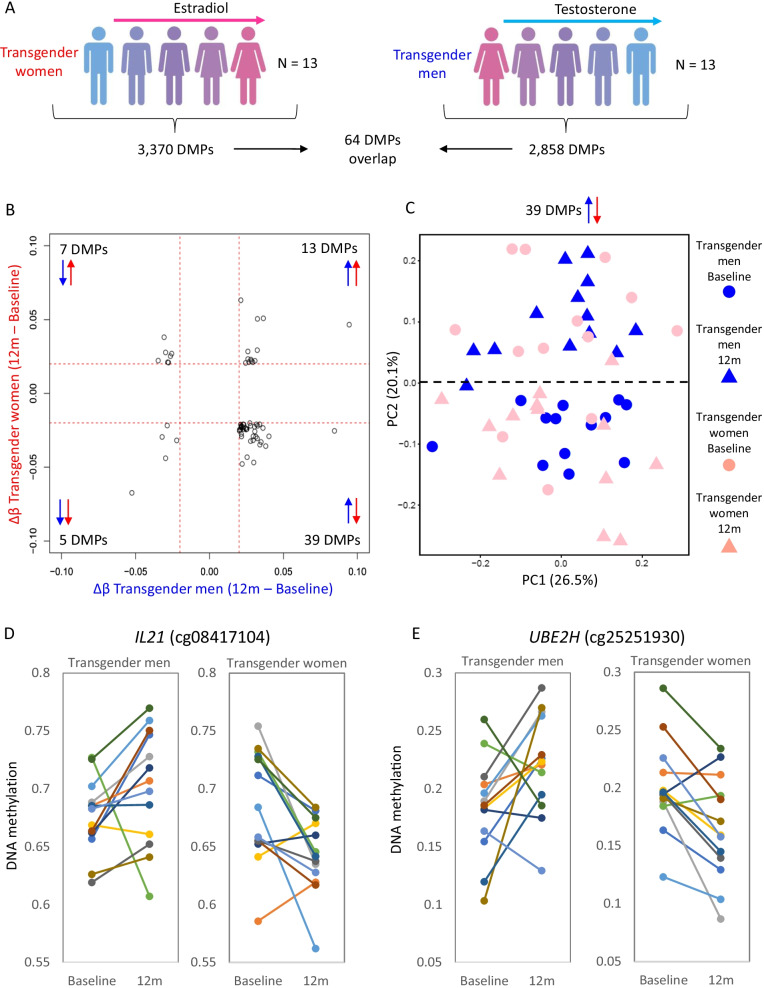
Little overlap in DMPs between transgender men on masculinizing GAHT and transgender women on feminizing GAHT. **A** Summary of DMPs identified in transgender women and transgender men comparison at *p* value < 0.05 and Δ*β* > 0.02. **B** Scatter plot of change in DNA methylation during GAHT (12 months - baseline) for transgender men (x axis) and transgender women (y axis) for 64 DMPs that are significant in both GAHT groups. The largest group of DMPs (39 in total) show higher DNA methylation after 12 months of masculinizing GAHT and lower DNA methylation after 12 months of feminizing GAHT. **C** PCA plot of individual samples at baseline and 12 months based on the 39 anti-correlating DMPs. PC2 separates 12 months from baseline samples, with transgender women moving downwards and transgender men moving upwards. **D** and **E** Dot plot showing DNA methylation level for individual donors at baseline and 12 months at top probes that show an inverse change in DNA methylation between transgender women and transgender men groups. The probes are associated with *IL21* and *UBE2H* genes

After 12 months GAHT, the majority of DMPs showed gains in DNA methylation in transgender men, whereas the majority of DMPs in transgender women showed loss of DNA methylation (Fig. 1B). Only a small overlap of 64 DMPs (1.9%) was found between feminizing and masculinizing GAHT comparisons (Fig. 2A, Additional file 6: Table S6). Of these, 46 showed a negative correlation, with 39 gaining DNA methylation in transgender men and losing methylation in transgender women (Fig. 2B). An example of this is an *IL21* promoter-associated DMP (cg08417104) which is similarly methylated in transgender women and transgender men at baseline, but gains DNA methylation after 12 months masculinizing GAHT (mean Δ*β* 0.025) and loses DNA methylation after 12 months feminizing GAHT (mean Δ*β*  -0.043) (Fig. 2D and 2E). Clustering of individual samples by PCA based on these 39 DMPs shows a shift of samples from transgender men on masculinizing GAHT towards that of GAHT-naïve individuals assigned male at birth (up on PC2 in Fig. 2C) and vice versa for samples from transgender women on feminizing GAHT (down on PC2 in Fig. 2C). This opposing direction of DNA methylation change is also evident when clustering samples based on all 5838 significant DMPs (unadjusted *p* value < 0.05, Δ*β* > 0.02) (Additional file 10: Fig. S5A).

### GAHT-associated DMPs are enriched at promoters of genes associated with anatomical development and immune processes

**Fig. 3:**
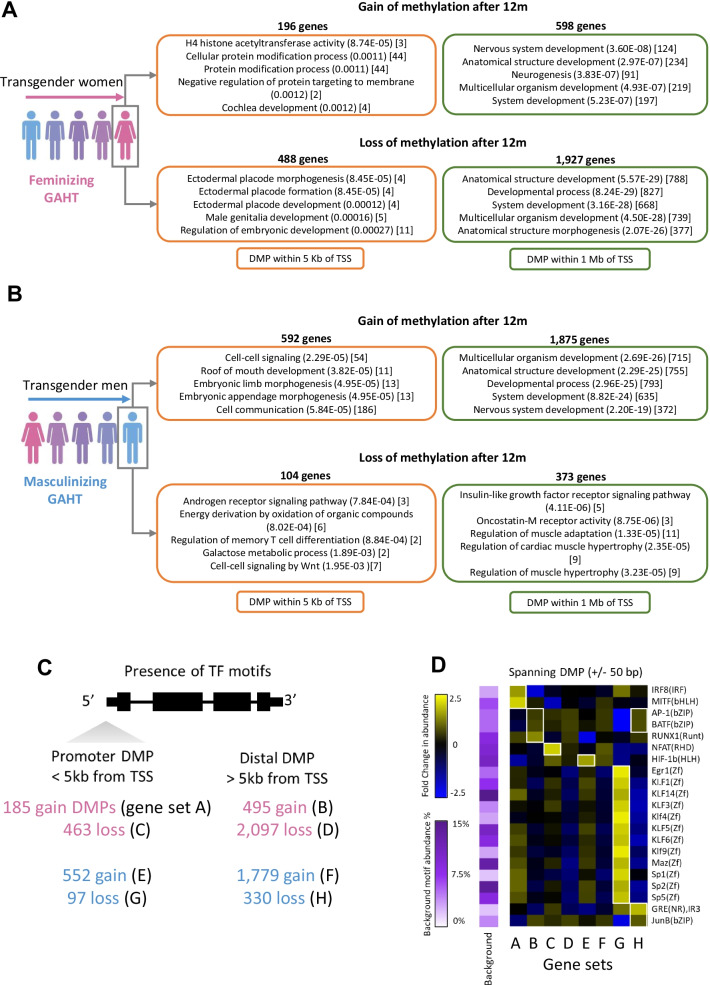
Genes with DMPs in promoters are enriched for biological processes and transcription factor binding motifs. **A** Top five most significantly enriched biological processes (BP) terms of genes with a DMP (transgender women 12 months vs baseline) within 5 kilobases (Kb) (in orange) or within 1 megabase (Mb) (in green) of a transcription start site (TSS). **B** Top five most significantly enriched BP terms of genes with a DMP (transgender men 12 months vs baseline) within 5 Kb or 1 Mb of a TSS. In **A** and **B**, BP terms are followed by (unadjusted *p* value) and [number of target genes in term]. Adjusted *p* values can be found in supplementary tables. **C** Outline of the number of 12-month GAHT-associated DMPs for feminizing (pink) or masculinizing (blue) GAHT, and whether DMP is promoter proximal (< 5 Kb of TSS) or distal (> 5 Kb of TSS). **D** Enriched transcription factor binding motifs (TFBMs) in genomic sequences spanning 50 bp upstream and downstream of promoter DMPs and distal DMPs. Heatmaps show the fold change (FC) in TFBM abundance relative to average background abundance (row-scaled), with white squares indicating TFBMs with unadjusted *p* value < 0.05, FC > 1.5, and change in abundance of > 5%

Genes with promoter-associated DMPs, defined as a DMP within 5 kilobases of a transcription start site (TSS), were involved in numerous biological processes relating to the immune response, cell signalling, metabolism, hormone signalling, and sex-associated anatomical processes, among others (Fig. 3A, B, Additional file 7: Table S7). In transgender women receiving feminizing GAHT, 488 genes had a loss-of-methylation promoter DMPs and 196 genes had a gain-of-methylation promoter DMPs at 12 months. In loss-of-methylation promoter DMPs, anatomical development processes were among the most enriched, including ‘ectodermal placode morphogenesis’ (unadjusted *p* value 8.45E−05) (comprised of *TBX3, HDAC1, TBX2,* and *CTNNB1*) and ‘male genitalia development’ (unadjusted *p* value 0.00016) (comprised of *BMP6, TBX3, LGR4, CTNNB1,* and *AR*). We also observed enrichment of numerous immune-related biological processes including ‘immune response’, ‘response to stimulus’, ‘lymphocyte-mediated immunity’, ‘positive regulation of NF-kB activity’, and ‘positive regulation of type I interferon production’ (Additional file 7: Table S7). When expanding gene lists to those with a DMP within 1 megabase of a transcription start site, we also observed strong enrichment of ‘anatomical structure development’ in both loss-of-methylation (unadjusted *p* value 5.57E−29) and gain-of-methylation (unadjusted *p* value 2.97E−07) gene lists (Fig. 3A, Additional file 7: Table S7).

In transgender men on masculinizing GAHT, 104 genes were associated with loss-of-methylation promoter DMPs, and 592 genes were associated with gain-of-methylation promoter DMPs at 12 months. The most enriched biological process in loss-of-methylation promoter DMP associated genes was ‘androgen receptor signalling pathway’ (unadjusted *p* value 7.84E−04) comprised of *DAXX, RWDD1,* and *PPARGC1A* (Fig. 3B)*.* Again we observed strong enrichment of anatomical related processes when using lists of genes associated with DMPs within 1 megabase of transcription start sites, with strong enrichment of ‘anatomical structure development’ ( unadjusted *p* value 2.29E−25) in gain-of-methylation DMP associated genes, and ‘regulation of muscle adaptation’ (unadjusted *p* value 1.33E−05) in loss-of-methylation DMP associated genes (Fig. 3B). The top 50 most significantly enriched (unadjusted *p* value < 0.05) biological processes and KEGG pathways of DMP associated genes can be found in Additional file 7: Table S7. These results suggest epigenetic remodelling at genomic loci associated with sex-associated anatomical changes, the immune response, and hormone signalling.

Further, in the comparison of our DMPs to a validated pregnancy-associated 15 probe DNA methylation signature, 3/15 were present in transgender women on feminizing GAHT and none in transgender men on masculinizing GAHT, reflecting the oestrogen-specific response (Additional file 10: Fig. S6A). Finally, we saw no overlap between our GAHT-associated DMPs and those identified in rheumatoid arthritis patients, a known sexually dimorphic autoimmune disease (Additional file 10: Fig. S6B).

Next, we scanned genomic regions surrounding promoter DMPs and distal DMPs for enrichment of transcription factor binding motifs (Fig. 3C). These provide insights into the potential regulatory mechanisms at regions with dynamic DNA methylation levels following GAHT. A motif was considered enriched if it had an unadjusted *p* value of < 0.05, showed an increase in abundance of > 5%, and a fold change in abundance of > 1.5 compared to background sequences. Generally, Egr1, Kruppel-like factor (KLF), and Sp family motifs showed higher abundance around promoter DMPs compared to distal DMPs, while AP-1 and BATF motifs were more abundant around distal DMPs (Fig. 3D). Some motifs showed exclusive enrichment in a particular group, including IRF8 and MITF motifs in gain-of-methylation promoter DMPs in transgender women and the NFAT motif in loss-of-methylation promoter DMPs in transgender women (Fig. 3D).

### Masculinizing GAHT DMRs include an age-associated sex-specific region in the promoter of PRR4

**Fig. 4:**
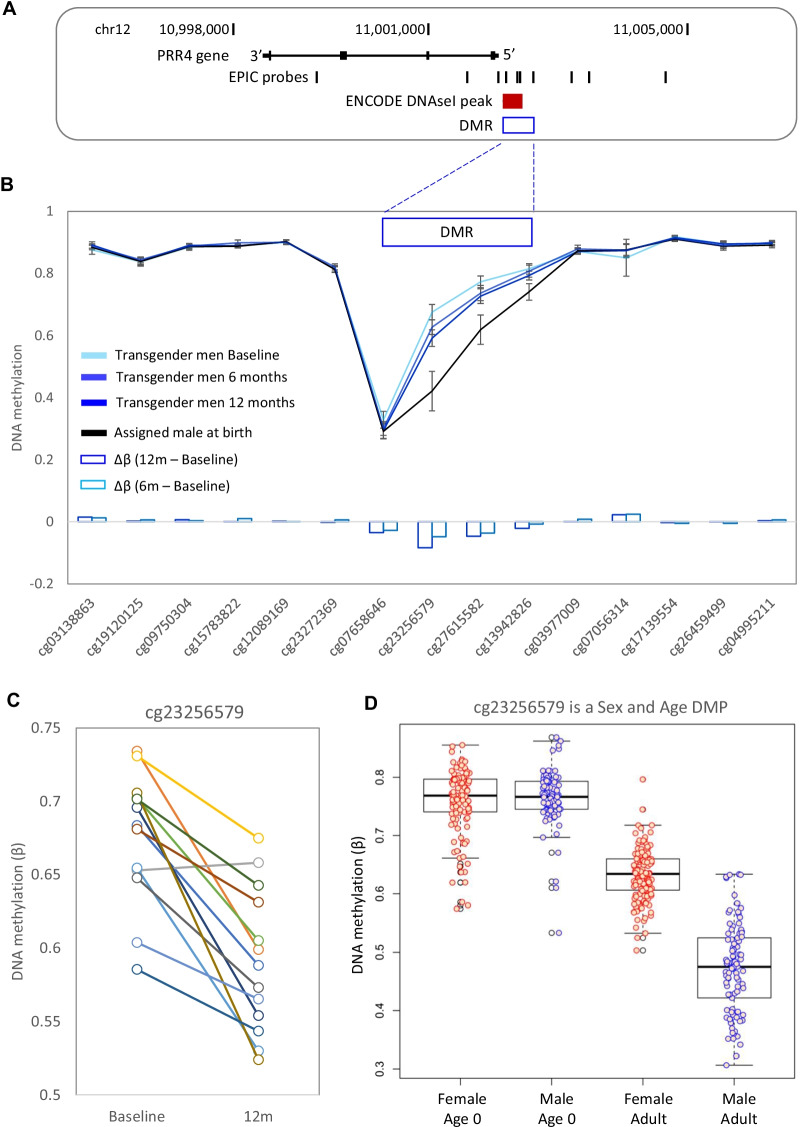
Detailed DNA methylation map of the *PPR4* gene. **A** Map of the *PRR4* gene in hg19, showing EPIC probe locations. **B** Mean DNA methylation level at *PRR4* for transgender men at baseline, transgender men at 12 months GAHT, and control individuals assigned male at birth. Error bars are 95% confidence intervals. Four DMPs within the DMR are losing DNA methylation in response to masculinizing GAHT, in the direction towards DNA methylation level typical of people assigned male at birth. **C** Line plot showing individual donor changes in DNA methylation for top probe within the *PRR4* DMR—cg23256579. **D** Boxplot of cg23256579 in blood at birth (Guthrie cards) and in adults (age 23–35), showing that sex-specific DNA methylation at this probe arises during the lifetime

Next, we examined differentially methylated regions (DMRs) which contain multiple DMPs that show correlative methylation (Additional file 10: Fig. S7A, Additional file 8: Table S8). We identified three DMRs in the transgender men group at 12 months (near *PRR4, PHF19,* and *MMP17*) (Additional file 10: Fig. S7B), with no DMRs detected in the transgender men group at 6 months. The *PRR4* DMR was in the promoter region, overlapped an ENCODE defined open chromatin region (Fig. 4A), and showed the largest change in DNA methylation relative to baseline, which was present at both 6 months and 12 months (Fig. 4, Additional file 10: Fig. S2F). To investigate this area further, we plotted the DNA methylation levels of probes in and around the DMR (Fig. 4B). This revealed that the region was sex-specific, showing lower DNA methylation in people assigned male at birth compared to people assigned female at birth (mean Δ*β* − 0.12 across the DMR). Masculinizing GAHT was associated with a loss of DNA methylation (mean Δ*β* − 0.03 across the DMR), shifting the DNA methylation pattern from that observed in people assigned female at birth towards that observed in people assigned male at birth (Fig. 4B). Within this DMR, cg23256579 exhibited the greatest loss of methylation following masculinizing GAHT, with 12 out of 13 donors showing robust loss of methylation (mean Δβ -0.084, *p* value 5.96E−05, Fig. 4C, Additional file 10: Fig. S2F). The promoter region of *PRR4* is known to be differentially methylated in an age-associated sex-specific manner, with people assigned male at birth starting to lose DNA methylation faster than people assigned female at birth during puberty [17, 24–27]. The sex-associated age-related methylation of cg23256579 was validated using publicly available data from another cohort (GSE131433, [28]) which is comprised of matched blood samples taken at birth (Guthrie cards) and in adulthood (venous blood collected at 22 to 35 years old). At this probe, we observed similar methylation levels at birth between people assigned male and people assigned female, with a loss of methylation in adult individuals assigned male compared to adult individuals assigned female (mean Δ*β* − 0.142, Fig. 4D).

### Feminizing GAHT-associated differentially methylated regions include an age-associated sex-specific region in the promoter of VMP1

**Fig. 5:**
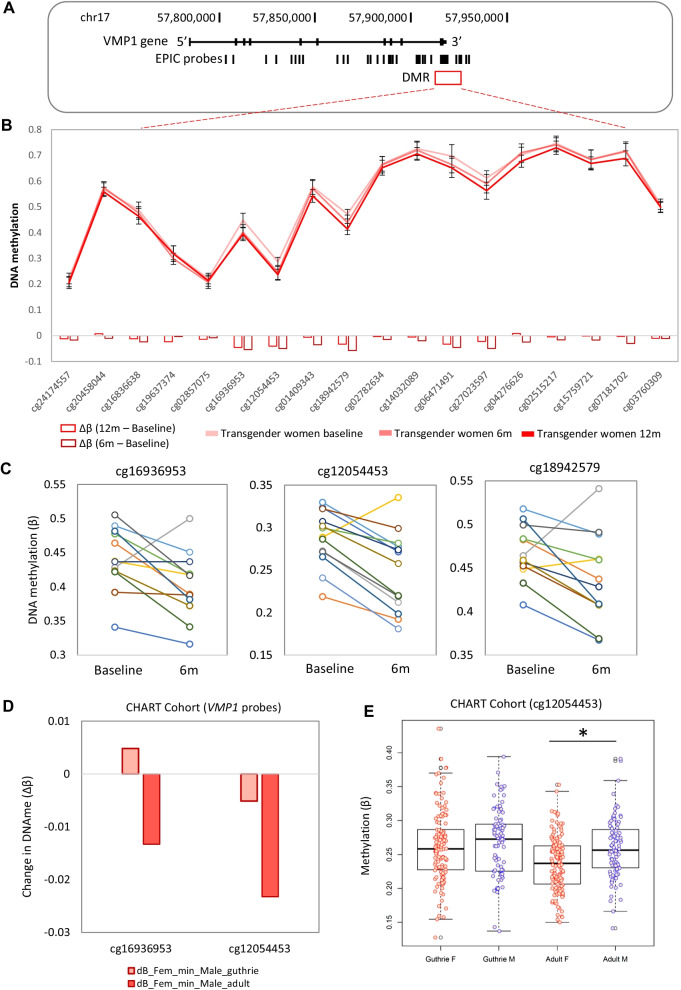
Detailed DNA methylation map of the *VMP1* gene. **A** Map of the *VMP1* gene in hg19, showing EPIC probe locations. **B** Mean DNA methylation level at *VMP1* for transgender women at baseline, 6 months GAHT, and 12 months GAHT. Error bars are 95% confidence intervals. Nine DMPs show the same direction of DNA methylation change within the DMR. **C** Line plot showing individual donor changes in DNA methylation for the top 3 probes within the *VMP1* DMR. **D** Bar plot showing difference in DNA methylation between people assigned female at birth and people assigned male at birth at the top 2 *VMP1* probes at birth and in adults. **E** Boxplot of cg12054453 in blood at birth (Guthrie cards) and in adults (23-35yo), showing that sex-specific DNA methylation at this probe become significant during the lifetime. *p value < 0.01

We identified five DMRs in transgender women at 6 months post-feminizing GAHT (near *TAS1R2, ZFP36L1, MED31, VMP1,* and *SLC35D3*) and three DMRs at 12 months post-feminizing GAHT (near *CAT, LANCL3,* and *HUS1*) (Additional file 10: Fig. S7D and Additional file 8: Table S8). A DMR at the 3’ UTR of *VMP1* (chr17:57,915,665-57,915,773, Fig. 5A, 5B) showed the largest change in DNA methylation relative to baseline, with 3 probes within this DMR (cg16936953, cg12054453, and cg18942579) being significantly less methylated at both 6 months and 12 months (Fig. 5C, Additional file 10: Fig. S2E, and Additional file 10: Fig. S7D). By analysing the broader region at the *VMP1* 3’ UTR, we identified eight probes that show lower DNA methylation after 6 months of feminizing GAHT and nine showing lower DNA methylation after 12 months of feminizing GAHT compared to baseline (Fig. 5B). The methylation levels of the top three probes in individual donors from baseline to 6 months are plotted in Fig. 5C (mean Δ*β* − 0.05, − 0.04, and − 0.03, respectively) and this loss of methylation was sustained at the 12-month time point (Additional file 10: Fig. S2E). The three probes also exhibit sex-associated methylation in our cohort at baseline, with participants assigned female at birth showing lower methylation than participants assigned male at birth (mean Δ*β* − 0.049, − 0.049, and − 0.032, respectively). The sex-associated methylation of two of these probes (cg16936953 and cg12054453) was validated using publicly available data (GSE131433) (Fig. 5D), and cg12054453 was also validated as a sex-associated age-related CpG showing similar methylation profiles between sexes at birth with a significant loss of methylation (*p* value < 0.01, mean Δ*β* − 0.023) in people assigned female at birth compared to people assigned male at birth by adulthood.

### X chromosome and autosomal sex-specific DNA methylation is largely unaffected by GAHT

**Fig. 6:**
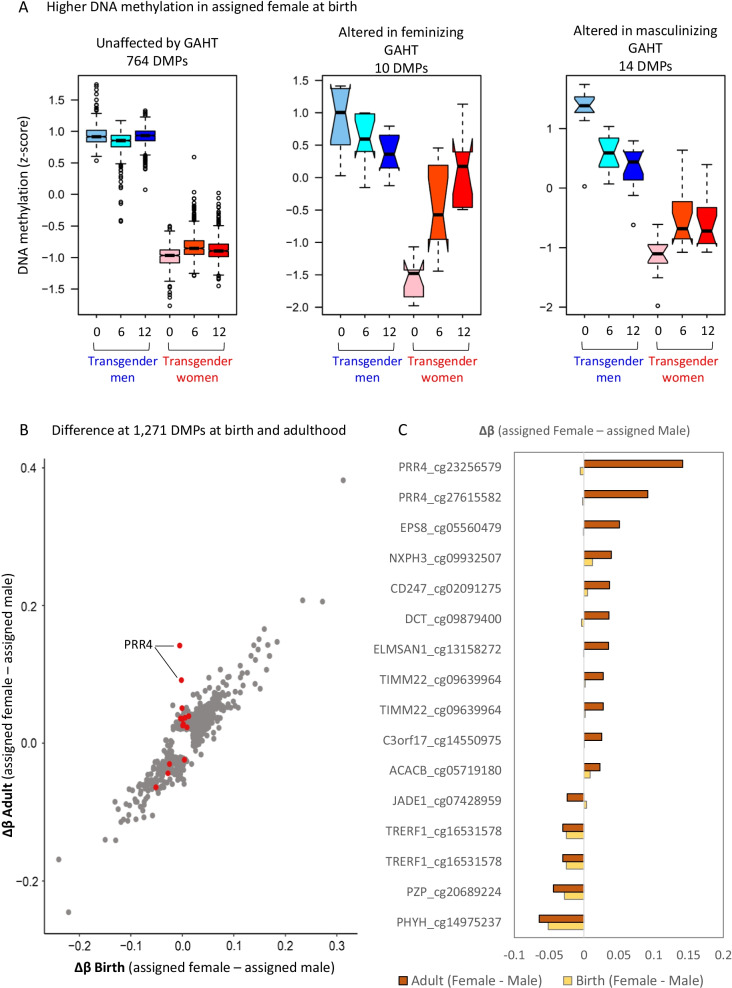
GAHT specifically influences age-associated sex-specific DMPs. **A** Boxplot of DNA methylation (z-scored) of sex-specific autosomal DMPs that show higher DNA methylation in people assigned female at birth compared to people assigned male at birth in our cohort (*p* value < 0.01, mean Δ*β* < − 0.05). See Additional file 10: Fig. S8 for X chromosome and autosomal DMPs with lower DNA methylation in people assigned female at birth. 764 DMPs are not affected by GAHT, while 10 and 14 DMPs are significant in the feminizing or masculinizing GAHT comparisons, respectively (mean Δ*β* > 0.02 or < − 0.02, *p* value < 0.05). **B** Scatter plot of change in DNA methylation (Δ*β*) between people assigned female and people assigned male at time of birth (Guthrie cards) (x axis) or in adulthood (y axis) based on a publicly available dataset (GSE131433). Red dots represent probes that are significant in transgender women or transgender men comparisons, grey dots are not significant. The red dots are generally different between sexes in adults, but not at birth. **C** Bar plot showing change in DNA methylation between sexes at probes shown in red in **B**, in adults (brown) and at birth (yellow)

Considering that the two top GAHT-induced DMRs, at *PRR4* and *VMP1*, were both age-associated sex-specific loci, we were interested to explore the overall effect of GAHT on sex-associated DNA methylation. By comparing GAHT-naïve transgender men and women (baseline samples), we identified 1271 sex-associated autosomal DMPs, with 788 showing higher methylation (*p* value < 0.01 and mean Δ*β* > 0.05) (Fig. 6A) and 483 showing lower methylation (Δ*β* < − 0.05) in individuals assigned female at birth compared to individuals assigned male at birth (Additional file 10: Fig. S8A). We then investigated whether these DMPs were affected by (mean Δ*β* < − 0.02, *p* value < 0.05) 12 months of masculinizing or feminizing GAHT, and whether this change reflected a transition towards the profile of the opposite sex. A total of 12,516 DMPs (mean Δ*β* > 0.05) were identified on the X chromosome, of which 98.9% were unaltered after 12 months of GAHT (Additional file 10: Fig. S8B), indicating that GAHT has little influence on X inactivation-associated DNA methylation patterns, including at the *XIST* locus (Additional file 10: Fig. S9). Of the 1271 sex-associated autosomal DMPs, 1228 (96.6%) were unaltered after 12 months of GAHT, while 19 (1.5%) were altered after feminizing GAHT and 24 (1.9%) were altered after masculinizing GAHT (Fig. 6A, Additional file 10: Fig. S8A, Additional file 9: Table S9). Of the 43 DMPs that were affected by GAHT, 41 showed a trend towards the methylation profile of the GAHT-naïve opposite sex (Additional file 10: Fig. S10). Among sex-associated autosomal DMPs affected by GAHT were probes in the promoter regions of *ACACB* (cg03619736), *HLA-DPB1* (cg00798281), and *PRR4* (cg23256579 and cg27615582) (Additional file 10: Fig. S10, Table S9). Further, the correlation between ‘assigned female at birth v assigned male at birth’ Δ*β* and ‘baseline v 12 m GAHT’ Δ*β* (Additional file 10: Fig. S5B, C), indicates that GAHT remodels DNA methylation towards the profile of the opposing sex.

We then tested the hypothesis that GAHT only influenced the DNA methylation level of CpG sites that become sex-specific over time, as was the case for *PRR4* and *VMP1* loci (Figs. 4 and 5). By correlating the change in DNA methylation between individuals assigned female at birth and individuals assigned male at birth at the 1271 sex-associated autosomal DMPs at birth (Guthrie cards) and in adulthood, we observe a strong correlation (Fig. 6B), indicating that most sex-specific DMPs are stable over time (grey dots). DMPs in response to GAHT are shown as red dots (Fig. 6B), with the majority showing no evidence of sex specificity at birth, in line with them being age-associated sex-specific DMPs. This was particularly true for DMPs that lose methylation in people assigned male at birth (Fig. 6C).

## Discussion

Gender-affirming hormone therapy is a cornerstone of transgender health care, but it is still not known whether GAHT influences immune function, susceptibility to autoimmune disease, or infection risk. With about 0.4% of the USA population identifying as transgender [29], of which 80% have either taken or are considering GAHT [30], it is important to understand how this treatment influences the molecular biology of immune cells [3]. Cells of the human hematopoietic lineage are sensitive to environmental exposures, which may influence their function through epigenetic remodelling [31–33]. Further, there is sexual dimorphism in cytokine responses [34], susceptibility to infection [35], and development of autoimmune disease [36]. A proportion of these effects are due to hormone-associated epigenetic remodelling, with periods of hormonal change, such as puberty [20, 21], pregnancy [37], and menopause HRT [38], associated with genome-wide DNA methylation changes in blood. In this study, we characterized longitudinal genome-wide DNA methylation changes in blood of transgender individuals undergoing GAHT (baseline, 6 months and 12 months post-GAHT). We demonstrate that both feminizing and masculinizing GAHT alter the blood methylome in a progressive manner (Fig. 1). This indicates that once established by 6 months, GAHT-associated DNA methylation changes are largely stable in the presence of hormone treatment.

It remains unclear whether transgender women and transgender men on GAHT retain the autoimmunity and infection risks of their sex assigned at birth. Case reports have indicated changes in autoimmunity following commencement of GAHT, including the improvement of subacute cutaneous lupus in transgender men receiving masculinizing hormone therapy [39] and onset of lupus or systemic sclerosis in transgender women following feminizing hormone therapy or sex reassignment surgery [40–43], suggesting a potential role of estradiol (or the absence of androgens) in the pathogenesis of autoimmune disorders. We found minimal overlap (0.1%) between GAHT-induced and the top five thousand rheumatoid arthritis-associated CpGs reported by Liu et al. (2013) (GEO dataset GSE42861, published in [44]) (Additional file 10: Fig. S6B), and no overlap with Sjogren’s- and SLE-associated CpGs [45] (data not shown). We did, however, find that gene promoters containing GAHT-associated DMPs are enriched for immune system processes, most markedly in genes that lose promoter DNA methylation during feminizing GAHT and genes that gain DNA methylation during masculinizing GAHT (Additional file 7: Table S7).

Transgender individuals are a highly marginalized population at higher risk of depression and antidepressant drug use [46], both of which have been associated with variation in blood DNA methylation patterns [47, 48]. A recent meta-analysis of more than 11,000 individuals identified 3 DMPs in blood associated with depression [49], but more longitudinal studies are required to explore this phenomenon further. Due to the small sample size, we were not able to explore the effect antidepressant use on GAHT-associated DNA methylation changes. Additionally, a higher chronic allostatic load in transgender individuals may affect their response to GAHT and the expression of inflammatory markers in the circulation [50, 51]. The present study involved longitudinal samples from transgender individuals without matched longitudinal cisgender controls. Future studies should include age-matched GAHT-free cisgender individuals to control for i) differences between the baseline methylation profile of transgender individuals compared to cisgender individuals and for ii) potential longitudinal changes that may be common across cisgender individuals and transgender individuals. Recently, Ramirez et al. (2021) identified 87 DMPs between cisgender men and transgender women prior to feminizing GAHT and 2 DMPs between cisgender women and transgender men prior to masculinizing GAHT [52].

In future studies, it would also be informative to investigate the effect of hormone blockers in younger transgender individuals and the effects of estradiol therapy alone compared to a combination of estradiol and progesterone or anti-androgens. Anti-androgen treatment was administered to 10 out of 13 transgender women in this study, but our sample size was too small to explore its effects compared to estradiol therapy only. Future studies with more interspersed time points and a longer follow-up period are also needed to fully characterize the stability and nature of the dynamic changes we observed at 6 months to 12 months following GAHT. Previously, Aranda et al*.* (2017) reported increased methylation of the androgen receptor (*AR*) promoter in transgender women after 12 months of feminizing hormone therapy and increased methylation of the oestrogen receptor 1 (*ESR1*) promoter in transgender men after 12 months of masculinizing hormones therapy [53].The same group showed that cisgender and transgender individuals display different levels of methylation at the *ESR1* promoter, which GAHT alters [54]. We did not observe DNA methylation changes at the *ESR1* promoter in our genome-wide data.

Our top DMRs were present at the promoter of *PRR4* and 3’ UTR region of *VMP1* (Figs. 4, 5). The proline-rich protein 4 (PRR4) is expressed in lacrimal glands and has been detected in a variety of other human tissues and cells (including CD4+ T cells, platelets, salivary glands, and skin). The biological function of PRR4 is not known, but *PRR4* has been shown to be downregulated in cancerous laryngeal tissue and in the tear fluid of pathological conditions of the eye [55]. Loss of methylation of the top probe in the DMR, cg23256579, was reported in peripheral blood cells and purified CD4+ T cells in people assigned female at birth with systemic lupus erythematosus (SLE) [56]. The *PRR4* DMR exhibits an age-associated sex-specific blood methylation pattern in adults [17, 24–27], with a recent study showing that the sex-specific loss of DNA methylation in people assigned male at birth occurs around 16 years of age [26]. This suggests that puberty-associated changes in circulating testosterone may be a potential driver of this sex-specific methylation. Our data shows that this testosterone-driven change can also be induced in adults, highlighting the epigenetic plasticity of the human immune system.

Vacuole membrane protein 1 (VMP1/TMEM49)*,* is involved in the formation of autophagosomes and in the interaction between the endoplasmic reticulum and other organelles [57]. Sex-specific differences in methylation at the *VMP1* DMR have been previously reported, also in an age-associated pattern [27, 58, 59]. Suderman et al. (2017) found that cg12054453 and cg16936953 show significant sex differences in methylation at age 17 (people assigned female showing lower methylation) but not at birth or age seven [26]. In agreement, we found that probes within this region show modest sex association, with people assigned female at birth showing a lower methylation than people assigned male at birth. After 6 months and 12 months of feminizing GAHT, transgender women exhibit a significant loss of methylation of approximately the same magnitude as the sex difference observed at baseline, thus resembling the methylation profile of people assigned female at birth (Fig. 5). One of the probes (cg16936953) has also been reported as progressively hypomethylated across pregnancy [37] (Additional file 10: Fig. S6), further supporting a role of female reproductive hormones, specifically estradiol, in inducing hypomethylation in pregnancy. In recent years, this region of *VMP1* has been consistently reported as hypomethylated in blood of patients with inflammatory bowel disease (IBD), Crohn’s disease (CD), and ulcerative colitis (UC) [60–62]. It is worth highlighting that the loss of methylation observed in IBD patients compared to healthy controls is more pronounced than the loss of methylation observed across feminizing GAHT [60, 61]. In childhood, people assigned female at birth have been shown to have a reduced risk of CD compared to people assigned male at birth (up until age 10–14), but then an increased risk after 25 years of age [63], suggesting a potential role of reproductive hormones in the sex bias of CD. Interestingly, hypogonadism has been observed in males with IBD at rates higher than expected [64], and testosterone therapy has been shown to have a positive effect in the clinical course of CD [65]. Moreover, Somineni et al*.* (2019) report that the methylation profiles of cg12054453 and cg16936953 in CD patients change after treatment to resemble methylation patterns observed in people without intestinal inflammation, and the authors conclude that CD-associated loss of methylation is likely a result of inflammation rather than CD pathogenesis. Nonetheless, the relationship between *VMP1* methylation, sex hormones, and the risk of CD warrants further investigation, as it is unclear whether transgender women are at higher risk of CD following GAHT.

## Conclusions

The GAHT model provided a unique opportunity to determine the proportion of previously published sex-specific differences on autosomal chromosomes that are sensitive to hormonal influence post-puberty. In total, GAHT affected 3% of all sex-specific autosomal DMPs, but with 15.8% of these previously reported as age-associated sex-specific DMPs (Fig. 6) [24]. This indicates that sex-specific DNA methylation that is present throughout life, from birth to adulthood, is hard-wired and determined by genetic and developmental programmes, and not susceptible to change in response to hormones. The second conclusion from this analysis is that puberty-associated changes in DNA methylation can be induced in adults. There are limitations to this study that are worth highlighting, such as the small sample size (*n* = 13 in each group), the poor overlap between 6-month and 12-month DMPs, and the lack of a replication in an independent dataset. Additionally, the sample type used in this study was buffy coat, and thus potential cell-specific changes in DNA methylation are masked. We recommend future studies to investigate DNA methylation changes in particular immune cell subsets. We measured cell composition using the ‘estimateCellCounts’ tool [66] and also performed analysis without cell composition in the model, but future studies may consider using additional cell composition tools [67–69]. Although the use of longitudinal samples allows for a reduced sample size, we did not have the power to look at the effect of age. Considering that we identified sex-associated age-related CpGs that are altered by GAHT, the magnitude of GAHT-induced effects may differ between age groups. There are several unexplored questions about the effect of GAHT, and from an epigenetic perspective, it would be interesting to explore if GAHT in adulthood, and puberty blockers in children and adolescents, influences ‘DNA methylation age’, a predictor of disease risk and all-cause mortality [70, 71]. Additionally, other molecular events, such as post-translational histone modifications and gene expression changes may be informative, especially for acute responses to GAHT. In summary, our study is the first epigenome-wide analysis of transgender men and transgender women during GAHT, and the observed epigenetic changes in blood warrant further cell-specific functional and molecular profiling. This study advances our understanding of the complex interplay between sex hormones, sex chromosomes, and DNA methylation in the context of immunity. We highlight the need to broaden the field of ‘sex-specific’ immunity beyond cisgender males and cisgender females to include transgender people, as we show that GAHT induces a unique molecular profile.

## Methods

### Study participants

Thirteen transgender men (assigned female at birth) seeking masculinizing GAHT and thirteen transgender women (assigned male at birth) seeking feminizing GAHT gave informed consent and consented to genetic testing of blood samples, as part of the project ‘The effects of cross-sex hormone therapy on bone microarchitecture in transgender individuals; a prospective controlled observational study’ based at the Austin Health (Human Research Ethics Committee project HREC/17/Austin/74). Median age at the commencement of GAHT was 23 years in transgender men , and 29 years in transgender women [22, 61].

### Gender-affirming hormone therapy regimen

Transgender men were taking full doses of either transdermal or intramuscular testosterone and transgender women were taking standard doses of either oral or transdermal estradiol as well as anti-androgen agents. Transgender individuals were recruited from endocrinology outpatient clinics and primary care general practice clinics specializing in transgender health in Melbourne, Australia.

All 13 transgender men received testosterone therapy (intramuscular (IM) testosterone undecanoate 1000 mg 12 weekly *n* = 7, IM testosterone enanthate (250 mg fortnightly) *n* = 1, transdermal testosterone gel 1% (1.25–5 g/day) *n* = 5). All 13 trans women received estradiol (oral estradiol valerate (dose range 1–8 mg daily) *n* = 5, or transdermal estradiol (25–200 mcg/24 h) *n* = 8. A total of 85% (*n* = 11) of the feminizing hormone therapy group were taking androgen blocking therapy in addition to estradiol therapy (cyproterone acetate 12.5 mg daily *n* = 6, spironolactone 100–200 mg daily *n* = 3, progesterone 100 mg daily *n* = 2). One participant (*n* = 1) had undergone orchiectomy since study enrolment. No individuals in the masculinizing hormone therapy group had undergone oophorectomy.

Estradiol was measured using immunoassay (Cobas E801, Roche Diagnostics, inter-assay variation 25% at level of 100 pnmol/L or less and 25% at a level of greater than 100 pmol/L). Testosterone was measured using electrochemiluminescent immunoassay (Cobas E801, Roche Diagnostics, inter-assay CV is 5.3% at a level of 3.4 nmol/L, 4.5% at 13.3 nmol/L, and 4.0% at 27.2 nmol/L). SHBG was measured on immunoassay (Cobas E801, Roche Diagnostics, inter-assay variation 6% at a level of 21 nmol/L and 6% at a level of 40 nmol/L). Adherence to hormone therapy regimens was evidenced by median sex hormone levels rising to within the affirmed gender reference range in both transgender men (serum testosterone reference range 10–35 nmol/L, 2.88–10.09 ng/mL) and transgender women (estradiol reference range 211–400 pmol/L, 57–109 pg/mL, serum testosterone reference range 2–4 nmol/L, 0.57–1.15 ng/mL) [72].

### Sample processing

Venous blood was collected at baseline (prior to or within 14 days of commencement of GAHT), and at 6 months and 12 months post-GAHT commencement. Part of the blood sample was sent for immediate analysis. The remaining blood sample was centrifuged, and the plasma component removed. An aliquot of the remaining buffy coat containing concentrated circulating blood cells was stored at − 30 °C.

### Genomic DNA extraction and DNA methylation profiling

Buffy coats were lysed with proteinase K for 2 h and the DNA was extracted using the Qiagen kit QIAamp® DNA Mini spin kit (Ref 56304) and eluted in 120 µL of elution buffer. Gel electrophoresis was used to confirm successful DNA extraction. Genomic DNA extracted from buffy coats was plated into 96-well plates at a concentration of 50 ng/µL (15 µL per well) and sent to Erasmus Medical Centre, Netherlands, for sodium bisulphite conversion and genome-wide methylation analysis using the Illumina Infinium Methylation EPIC Array, which measures DNA methylation across 850,000 CpG sites (referred to as EPIC ‘probes’), spanning promoter regions, gene bodies, and ENCODE-assigned distal regulatory elements. The output of the EPIC array is beta (*β*) values for each probe, which range from 0 to 1.

### DNA methylation data cleaning and normalization

Raw IDAT files for were processed using the MissMethyl and minfi packages for R [73, 74]. All samples had a good quality score (mean detection *p* value of < 0.01). Data were normalized for both within and between array technical variation using SWAN (Subset-quantile Within Array Normalization) [75]. We additionally performed analysis using M values normalized using Funnorm [76] to ensure that all results we discuss can be replicated using an alternative approach. Probes with poor average quality scores (detection *p* value > 0.01), those that overlap a SNPs at their CG site, and cross-reactive probes were removed from further analysis [77]. This left a total of 787,296 probes for downstream analysis. Cell composition was determined using the estimateCellCounts tool, with the ‘Blood’ reference dataset [66].

### Identification of GAHT-associated differentially methylated probes

Differential methylation analysis by linear regression modelling was performed on M values using limma’s lmFit function for model fitting and eBayes function for empirical Bayes analysis [78]. The relationship between M value and B value is *M* = log2(*B*/(1−*B*)), as previously described [79]. The M value is used to obtain normality in response variable to satisfy linear modelling assumptions. Confounders and covariates were identified using principal component analysis (shown in Additional file 10: Fig. S1). The models for comparing sample mean methylation (M value) at each probe between time points (Feminizing baseline v 6 m, baseline v 12 m, and Masculinizing baseline v 6 m, and baseline v 12 m) included: an indicator for time point, cell composition (CD8T + CD4T + NK + Bcell + Neutrophils), technical covariates (Sentrix_ID + Sample_well + Sentrix_position), and donor ID. Analysis was additionally performed without cell composition data as a covariate (Time point + Sentrix_ID + Sample_well + Sentrix_position + donor ID) and including age as a covariate (Time point + CD8T + CD4T + NK + Bcell + Neutrophils + Sentrix_ID + Sample_well + Sentrix_position + donor ID + age). The donor was modelled as a fixed effect, but we confirmed that probes remain significant when the donor was modelled as a random effect in mixed-effects models using the duplicateCorrelation function in limma. The limma output was then merged with a table of mean beta values. Differentially methylated probes (DMPs) were those with an unadjusted *p* value of < 0.05 and a change in methylation (delta beta or Δ*β*) of ≥ 0.02 (Additional file 1: Table S1 and Additional file 2: Table S2). These tables include the limma output, the mean DNA methylation for each group: Transwomen baseline, transwomen 6 m, transwomen 12 m, transmen baseline, transmen 6 m and transmen 12 m, and additional columns stating if the DMP was significant after Funnorm normalization, addition of age as a covariate, removal of cell composition as covariates. In order to identify temporal DMPs, k-means clustering was performed using MultiExperiment Viewer (MeV) [80], which groups DMPs into clusters with the nearest mean over time. This approach identified transient or progressive DMPs over time, as previously described [81]. Finally, sex-specific probes were identified as those that were significantly different between assigned males and assigned females at *p* value < 0.01, Δ*β* > 0.05. These were further separated into those that occur on the X chromosome and those on autosomes.

DMPs were assigned to the nearest gene within 1 megabase (1 Mb) using the GREAT tool [82]. Differentially methylated regions (DMRs) were identified using the DMRcate tool (version 2.8.0) [83] with *p* value < 0.05, and a minimum of 3 CpGs with at least one containing a Δ*β* of > 0.02 or < − 0.02. Bedtools was used to intersect DMRs with individual probes [84].

### Publicly available DNA methylation data

To put our findings into context, we used previously generated EPIC array data from age-specific sex-associated profiles GSE71245 [16], GSE60275 [85], GSE67393 [86], and rheumatoid arthritis GSE42861 [44].

### Motif enrichment and gene ontology analysis

DMPs were mapped to the closest gene using GREAT (http://great.stanford.edu/public/html/) and gene promoters were scanned for enriched transcription factor binding motifs (TFBMs) using the HOMER findMotifs tool (http://homer.ucsd.edu/homer/) [87]. DNA sequences spanning DMPs (± 50 base pairs) were also scanned for enriched motifs using the HOMER findMotifsGenome tool. HOMER scans for 440 validated motifs and the output include the frequency of the motif in the test regions and a p value (determined by hypergeometric test). Motifs were considered enriched if they had a p value < 0.05,an increase in motif frequency of > 5% compared to a background set of regions (% targets sequences containing motif—% background sequences containing motif) and a fold change in frequency of > 1.5 (% target sequences containing motif / % background sequences containing motif), as previously described [81]. To gain insight into biological function of genes associated with DMPs, enriched biological processes (BP) and KEGG pathways, were also identified using HOMER (HOMER scans for enrichment of 16,577 BP terms and 502 KEGG pathway terms and returns an unadjusted *p* value (determined by hypergeometric test)). The nearest gene to the DMP was the input for the hypergeometric analysis. DMPs were selected only by an unadjusted *p* < 0.05. Using a more restrictive DMP set with a lower selected p value cut-off could potentially change the gene ontology results. In addition to the HOMER unadjusted *p* value for pathway enrichment, we added a Benjamini–Hochberg corrected *p* value in the supplementary tables (Additional file 3: Table S3 and Additional file 7: Table S7).

## Supplementary Information

**Additional file 1: Table S1**. DMPs in transgender women (TW) on feminizing GAHT 6-month or 12-month comparison.

**Additional file 2: Table S2**. DMPs in transgender men (TM) on masculinizing GAHT 6-month or 12-month comparison.

**Additional file 3: Table S3**. Gene Ontology terms for genes associated with DMPs in each k-means cluster shown in Figure S4.

**Additional file 4: Table S4**. DMPs in transgender women (TW) on feminizing GAHT using the timecourse model.

**Additional file 5: Table S5**. DMPs in transgender men (TM) on masculinizing GAHT using the timecourse model.

**Additional file 6: Table S6**. 64 DMPs that overlap between transgender women and transgender men 12-month comparisons.

**Additional file 7: Table S7**. Gene Ontology terms for 12m feminizing or masculinizing GAHT, using genes within 1Mb of a DMP or those with a DMP in their promoter (5kb of a DMP).

**Additional file 8: Table S8**. Differentially methylated regions (DMRs) in transgender women and transgender men comparisons.

**Additional file 9: Table S9**. Sex-specific DMPs affected by GAHT.

**Additional file 10: Fig. S1**. Summary of covariates used in the EWAS analysis. **A**. PCA loadings in **B**. Heatmap highlighting the contribution of different variables to each principal component. Technical variation, such as location of sample in the EPIC array, was the biggest contributor to variation, followed by cell composition. The top number in each box within the heatmap is the correlation between principal component and data traits and underneath in brackets are the p values. Covariates with a high correlation and low p value are highlighted in red for positive correlations and blue for negative correlations. **Fig. S2**. **A**. Bar plot showing change in DNA methylation (Δβ) relative to baseline for top DMPs in the baseline vs 6 months or baseline vs 12 months comparison for transgender women. **B**. Bar plot showing change in DNA methylation (Δβ) or top DMPs in transgender men. **C**-**F**. Line plot showing DNA methylation changes per donor (different colour per donor) over time for selected DMPs. The mean DNA methylation level is shown as a bar plot. The X-axis refers to the GAHT group and time: TW = Transgender women, TM = Transgender men. **Fig. S3**. DNA methylation signature separates baseline from 6-month and 12-month GAHT samples. **A**. Scatter plot of change in DNA methylation relative to baseline for DMPs that are significant at both 6m and 12m following feminizing GAHT. There is a positive correlation between 6m-baseline Δβ (x axis) and 12m-baseline Δβ (y axis). **B**. Scatter plot of change in DNA methylation relative to baseline for DMPs that are significant at both 6m and 12m following masculinizing GAHT. There is a positive correlation between 6m-baseline Δβ (x axis) and 12m-baseline Δβ (y axis). **C**. Direction-of-change Venn diagram depicting overlap of 6m and 12m gain or loss of methylation DMPs in transgender women (TW). **D**. Direction-of-change Venn diagram depicting overlap of 6m and 12m gain or loss of methylation DMPs in transgender men (TM). In **C**. and **D**., we observe that overlapping DMPs show the same pattern of change (gain or loss). **E**. PCA Plot showing separation of samples based on DMPs in transgender women. A clear separation of 6m and 12m samples from baseline is observed on PC3. **F**. PCA Plot showing separation of samples based on DMPs in transgender men. A clear separation of 6m and 12m samples from baseline is observed on PC2. **Fig. S4**. DMP clusters identify temporal DNA methylation dynamics during GAHT. **A**. Boxplots showing DNA methylation level (z-score) for feminizing GAHT DMP clusters. **B**. Boxplots showing DNA methylation level (z-score) for masculinizing GAHT DMP clusters. K-means clustering identified 8 feminizing GAHT clusters and 6 masculinizing GAHT clusters. The dynamic nature of each cluster is labelled at the top of each boxplot and the number of DMPs in each cluster is displayed under the cluster description. The gene ontology terms associated with these clusters are shown in **Table S3**, while the DMPs that belong to each cluster are labelled in **Table S1** and **S2**. TM = transgender men, TW = transgender women. **Fig. S5**. GAHT-associated DNA methylation change generally trends towards the profile of opposite assigned sex. **A**. PCA plot based on all DMPs identified in transgender women or transgender men comparisons. Individual donors are shown, as are the trajectory of group averages (open circles and triangles). In general, the two GAHT groups move in opposite directions on PC2, similarly to the pattern observed when only the 39 anti-correlating DMPs are used in **Fig. S2**. **B**. Scatter plot showing correlation between change in DNA methylation in transgender women 12m – baseline (x axis) and people assigned female at birth – people assigned male at birth (y axis). **C**. Scatter plot showing correlation between change in DNA methylation in transgender men at 12m – baseline (x axis) and people assigned male at birth – people assigned female at birth (y axis). **Fig. S6**. Overlap between GAHT DMPs and pregnancy and autoimmune disease-related DMPs. **A**. Overlap between GAHT DMPs identified in our study and a pregnancy cohort. Out of a total of 15 validated pregnancy-associated DMPs, 3 (20%) are significant in the feminizing GAHT group, while none are significant in the masculinizing GAHT group. This confirms that these DMPs are sensitive to oestrogen in both GAHT and third trimester pregnancy. **B**. Overlap between GAHT DMPs and rheumatoid arthritis-associated DMPs, only a 0.1% overlap was identified. **Fig. S7**. Summary of Differentially Methylated Regions (DMRs) associated with GAHT. **A**. Strategy for identifying DMRs using DMRcate. **B**. Summary of DMRs identified at 6 months and 12 months following GAHT in feminizing and masculinizing groups. **C**. Bar plots of 12-month DMRs in transgender men. Bar plots show change in DNA methylation (Δβ) after 6 months (light blue) and 12 months (dark blue) of masculinizing GAHT relative to baseline in transgender men. **D**. Bar plots of 6-month DMRs in transgender women. Bar plots show change in DNA methylation (Δβ) after 6 months (pink) and 12 months (red) of feminizing GAHT relative to baseline in transgender women. **E**. Bar plots of 12-month DMRs in transgender women. No DMRs were identified in the 6-month comparison in transgender men. **Fig. S8**. Effect of GAHT on sex-specific DNA methylation patterns on autosomes and X chromosome. Boxplots of DNA methylation (z-scored) of sex-specific autosomal DMPs (**A**), and X chromosome DMPs (baseline transgender men vs baseline transgender women Δβ > 0.05 or < -0.05 and p value <0.01) (**B** and **C**). The first panels (left) are DMPs that are not influenced by GAHT, while centre and right panels are DMPs that are significant in transgender women and transgender men 12-month vs baseline comparisons, respectively (Δβ > 0.02 or < -0.02, and p value < 0.05). **Fig. S9**. Effect of GAHT on DNA methylation levels at the *XIST* locus. Bar plot shows the mean level of DNA methylation in transgender women and transgender men at baseline, 6 months, and 12 months into GAHT. Both feminizing and masculinizing GAHT had no effect on XIST DNA methylation. **Fig. S10**. Direction of change at sex-specific DMPs that are affected by GAHT. Bar plots showing change in DNA methylation (Δβ) for (**A**) sex-specific DMPs that show gain of methylation in people assigned female at birth (red) and are significant in the transgender men (12-month masculinizing GAHT) comparison (blue) and (**B**) sex-specific DMPs that show gain of methylation in people assigned female at birth and are significant in the transgender women (12-month feminizing GAHT) comparison (pink). **C**. Change in DNA methylation (Δβ) for sex-specific DMPs that show loss of methylation in people assigned female at birth and are significant in the transgender men (12-month masculinizing GAHT) comparison and (**D**) sex-specific DMPs that show loss of methylation in people assigned female and are significant in the transgender women (12-month feminizing GAHT) comparison. Common probes are highlighted, with the corresponding gene name.

## Data Availability

The genome-wide DNA methylation dataset produced in this study is available at Gene Expression Omnibus GSE176394 (https://www.ncbi.nlm.nih.gov/geo/query/acc.cgi?acc=GSE176394).

## References

[1] Moyer AM, Matey ET, Miller VM. Individualized medicine: Sex, hormones, genetics, and adverse drug reactions. Pharmacol Res Perspect. 2019;7(6):e00541-e. 10.1002/prp2.541 PMC6897337 31844524

[2] Short SE, Yang YC, Jenkins TM. Sex, gender, genetics, and health. Am J Public Health. 2013;103 Suppl 1(Suppl 1):S93-S101. 10.2105/AJPH.2013.301229 PMC3786754 23927517

[3] Dotto G-P (2019). Gender and sex-time to bridge the gap. EMBO Mol Med.

[4] Klein SL, Flanagan KL (2016). Sex differences in immune responses. Nat Rev Immunol.

[5] Markle JG, Fish EN (2014). SeXX matters in immunity. Trends Immunol.

[6] Selmi C, Gershwin ME (2019). Sex and autoimmunity: proposed mechanisms of disease onset and severity. Expert Rev Clin Immunol.

[7] Bouman A, Heineman MJ, Faas MM (2005). Sex hormones and the immune response in humans. Hum Reprod Update.

[8] Bereshchenko O, Bruscoli S, Riccardi C. Glucocorticoids, Sex Hormones, and Immunity. 2018;9(1332). 10.3389/fimmu.2018.01332 PMC6006719 29946321

[9] Shepherd R, Cheung AS, Pang K, Saffery R, Novakovic B. Sexual Dimorphism in Innate Immunity: The Role of Sex Hormones and Epigenetics. 2021;11(3559). 10.3389/fimmu.2020.604000 PMC7873844 33584674

[10] Jones PA (2012). Functions of DNA methylation: islands, start sites, gene bodies and beyond. Nat Rev Genet.

[11] Smith ZD, Chan MM, Humm KC, Karnik R, Mekhoubad S, Regev A (2014). DNA methylation dynamics of the human preimplantation embryo. Nature.

[12] Feinberg AP (2018). The key role of epigenetics in human disease prevention and mitigation. N Engl J Med.

[13] Heard E, Clerc P, Avner P (1997). X-chromosome inactivation in mammals. Annu Rev Genet.

[14] Weber M, Hellmann I, Stadler MB, Ramos L, Paabo S, Rebhan M (2007). Distribution, silencing potential and evolutionary impact of promoter DNA methylation in the human genome. Nat Genet.

[15] Gabory A, Attig L, Junien C (2009). Sexual dimorphism in environmental epigenetic programming. Mol Cell Endocrinol.

[16] Mamrut S, Avidan N, Staun-Ram E, Ginzburg E, Truffault F, Berrih-Aknin S (2015). Integrative analysis of methylome and transcriptome in human blood identifies extensive sex- and immune cell-specific differentially methylated regions. Epigenetics.

[17] Inoshita M, Numata S, Tajima A, Kinoshita M, Umehara H, Yamamori H (2015). Sex differences of leukocytes DNA methylation adjusted for estimated cellular proportions. Biol Sex Differ.

[18] Hall E, Volkov P, Dayeh T, Esguerra JL, Salo S, Eliasson L (2014). Sex differences in the genome-wide DNA methylation pattern and impact on gene expression, microRNA levels and insulin secretion in human pancreatic islets. Genome Biol.

[19] Gruzieva O, Merid SK, Chen S, Mukherjee N, Hedman AM, Almqvist C, et al. DNA methylation trajectories during pregnancy. Epigenet insights. 2019;12:2516865719867090-. 10.1177/2516865719867090 PMC6696836 31453433

[20] Almstrup K, Johansen ML, Busch AS, Hagen CP, Nielsen JE, Petersen JH (2016). Erratum: Pubertal development in healthy children is mirrored by DNA methylation patterns in peripheral blood. Sci Rep.

[21] Thompson EE, Nicodemus-Johnson J, Kim KW, Gern JE, Jackson DJ, Lemanske RF (2018). Global DNA methylation changes spanning puberty are near predicted estrogen-responsive genes and enriched for genes involved in endocrine and immune processes. Clin Epigenetics.

[22] Cheishvili D, Parashar S, Mahmood N, Arakelian A, Kremer R, Goltzman D (2018). Identification of an epigenetic signature of osteoporosis in blood DNA of postmenopausal women. J Bone Miner Res.

[23] Ronkainen PH, Pöllänen E, Alén M, Pitkänen R, Puolakka J, Kujala UM (2010). Global gene expression profiles in skeletal muscle of monozygotic female twins discordant for hormone replacement therapy. Aging Cell.

[24] Yusipov I, Bacalini MG, Kalyakulina A, Krivonosov M, Pirazzini C, Gensous N, et al. Age-related DNA methylation changes are sex-specific: a comprehensive assessment. bioRxiv. 2020:2020.01.15.905224. 10.18632/aging.202251 PMC7762479 33276343

[25] Vershinina O, Bacalini MG, Zaikin A, Franceschi C, Ivanchenko M (2021). Disentangling age-dependent DNA methylation: deterministic, stochastic, and nonlinear. Sci Rep.

[26] Suderman M, Simpkin A, Sharp G, Gaunt T, Lyttleton O, McArdle W, et al. Sex-associated autosomal DNA methylation differences are wide-spread and stable throughout childhood. bioRxiv. 2017:118265.

[27] McCartney DL, Zhang F, Hillary RF, Zhang Q, Stevenson AJ, Walker RM (2019). An epigenome-wide association study of sex-specific chronological ageing. Genome medicine.

[28] Novakovic B, Lewis S, Halliday J, Kennedy J, Burgner DP, Czajko A (2019). Assisted reproductive technologies are associated with limited epigenetic variation at birth that largely resolves by adulthood. Nat Commun.

[29] Meerwijk EL, Sevelius JM (2017). Transgender population size in the United States: a meta-regression of population-based probability samples. Am J Public Health.

[30] Nguyen HB, Chavez AM, Lipner E, Hantsoo L, Kornfield SL, Davies RD (2018). Gender-affirming hormone use in transgender individuals: impact on behavioral health and cognition. Curr Psychiatry Rep.

[31] Divangahi M, Aaby P, Khader SA, Barreiro LB, Bekkering S, Chavakis T (2021). Trained immunity, tolerance, priming and differentiation: distinct immunological processes. Nat Immunol.

[32] Lau CM, Adams NM, Geary CD, Weizman OE, Rapp M, Pritykin Y (2018). Epigenetic control of innate and adaptive immune memory. Nat Immunol.

[33] Zhang Q, Cao X (2019). Epigenetic regulation of the innate immune response to infection. Nat Rev Immunol.

[34] Ter Horst R, Jaeger M, Smeekens SP, Oosting M, Swertz MA, Li Y, et al. Host and Environmental Factors Influencing Individual Human Cytokine Responses. Cell. 2016;167(4):1111–24 e13. 10.1016/j.cell.2016.10.018 PMC5787854 27814508

[35] Zuk M (2009). The sicker sex. PLoS Pathog.

[36] Rubtsova K, Marrack P, Rubtsov AV (2015). Sexual dimorphism in autoimmunity. J Clin Invest.

[37] Gruzieva O, Merid SK, Chen S, Mukherjee N, Hedman AM, Almqvist C (2019). DNA methylation trajectories during pregnancy. Epigenet Insights.

[38] Bahl A, Pöllänen E, Ismail K, Sipilä S, Mikkola T, Berglund E (2015). Hormone Replacement Therapy Associated White Blood Cell DNA Methylation and Gene Expression are Associated With Within-Pair Differences of Body Adiposity and Bone Mass.

[39] Ocon A, Peredo-Wende R, Kremer JM, Bhatt BD (2017). Significant symptomatic improvement of subacute cutaneous lupus after testosterone therapy in a female-to-male transgender subject. Lupus.

[40] Zandman-Goddard G, Solomon M, Barzilai A, Shoenfeld Y (2007). Lupus erythematosus tumidus induced by sex reassignment surgery. J Rheumatol.

[41] Chan KL, Mok CC (2013). Development of systemic lupus erythematosus in a male-to-female transsexual: the role of sex hormones revisited. Lupus.

[42] Pontes LT, Camilo DT, De Bortoli MR, Santos RSS, Luchi WM (2018). New-onset lupus nephritis after male-to-female sex reassignment surgery. Lupus.

[43] Campochiaro C, Host LV, Ong VH, Denton CP (2018). Development of systemic sclerosis in transgender females: a case series and review of the literature. Clin Exp Rheumatol.

[44] Liu Y, Aryee MJ, Padyukov L, Fallin MD, Hesselberg E, Runarsson A (2013). Epigenome-wide association data implicate DNA methylation as an intermediary of genetic risk in rheumatoid arthritis. Nat Biotechnol.

[45] Imgenberg-Kreuz J, Almlof JC, Leonard D, Sjowall C, Syvanen AC, Ronnblom L (2019). Shared and unique patterns of DNA methylation in systemic lupus erythematosus and primary Sjogren's Syndrome. Front Immunol.

[46] Zwickl S, Wong AFQ, Dowers E, Leemaqz SY, Bretherton I, Cook T (2021). Factors associated with suicide attempts among Australian transgender adults. BMC Psychiatry.

[47] Li M, D'Arcy C, Li X, Zhang T, Joober R, Meng X (2019). What do DNA methylation studies tell us about depression? A systematic review. Transl Psychiatry.

[48] Lisoway AJ, Zai CC, Tiwari AK, Kennedy JL (2018). DNA methylation and clinical response to antidepressant medication in major depressive disorder: a review and recommendations. Neurosci Lett.

[49] Story Jovanova O, Nedeljkovic I, Spieler D, Walker RM, Liu C, Luciano M (2018). DNA methylation signatures of depressive symptoms in middle-aged and elderly persons: meta-analysis of multiethnic epigenome-wide studies. JAMA Psychiat.

[50] Cohen M, Karrington B, Trachtman H, Salas-Humara C. Allostatic Stress and Inflammatory Biomarkers in Transgender and Gender Expansive Youth: Protocol for a Pilot Cohort Study. JMIR Res Protoc. 2021;10(5):e24100. 10.2196/24100 PMC8173394 34009131

[51] Juster RP, de Torre MB, Kerr P, Kheloui S, Rossi M, Bourdon O (2019). Sex differences and gender diversity in stress responses and allostatic load among workers and LGBT people. Curr Psychiatry Rep.

[52] Ramirez K, Fernández R, Collet S, Kiyar M, Delgado-Zayas E, Gómez-Gil E, et al. Epigenetics Is Implicated in the Basis of Gender Incongruence: An Epigenome-Wide Association Analysis. Front Neurosci. 2021;15:701017. 10.3389/fnins.2021.701017 PMC8418298 34489625

[53] Aranda G, Fernández-Rebollo E, Pradas-Juni M, Hanzu FA, Kalko SG, Halperin I (2017). Effects of sex steroids on the pattern of methylation and expression of the promoter region of estrogen and androgen receptors in people with gender dysphoria under cross-sex hormone treatment. J Steroid Biochem Mol Biol.

[54] Fernández R, Ramírez K, Gómez-Gil E, Cortés-Cortés J, Mora M, Aranda G (2020). Gender-affirming hormone therapy modifies the CpG methylation pattern of the ESR1 gene promoter after six months of treatment in transmen. J Sex Med.

[55] Ekizoglu S, Ulutin T, Guliyev J, Buyru N (2018). PRR4: a novel downregulated gene in laryngeal cancer. Oncol Lett.

[56] Mok A, Solomon O, Nayak RR, Coit P, Quach HL, Nititham J, et al. Genome-wide profiling identifies associations between lupus nephritis and differential methylation of genes regulating tissue hypoxia and type 1 interferon responses. Lupus Sci Med. 2016;3(1):e000183-e. 10.1136/lupus-2016-000183 PMC5174796 28074145

[57] Wang P, Kou D, Le W. Roles of VMP1 in Autophagy and ER–Membrane Contact: Potential Implications in Neurodegenerative Disorders. 2020;13(42). 10.3389/fnmol.2020.00042 PMC7137732 32296305

[58] Yu C, Wong EM, Joo JE, Hodge AM, Makalic E, Schmidt D, et al. Epigenetic drift association with cancer risk and survival, and modification by sex. Cancers. 2021;13(8). 10.3390/cancers13081881 PMC8070898 33919912

[59] Yusipov I, Bacalini MG, Kalyakulina A, Krivonosov M, Pirazzini C, Gensous N (2020). Age-related DNA methylation changes are sex-specific: a comprehensive assessment. Aging (Albany NY).

[60] Adams AT, Kennedy NA, Hansen R, Ventham NT, OʼLeary KR, Drummond HE, et al. Two-stage genome-wide methylation profiling in childhood-onset Crohn's Disease implicates epigenetic alterations at the VMP1/MIR21 and HLA loci. Inflamm Bowel Dis. 2014;20(10):1784–93. 10.1097/MIB.0000000000000179 PMC4736293 25144570

[61] Ventham NT, Kennedy NA, Adams AT, Kalla R, Heath S, O'Leary KR (2016). Integrative epigenome-wide analysis demonstrates that DNA methylation may mediate genetic risk in inflammatory bowel disease. Nat Commun.

[62] Somineni HK, Venkateswaran S, Kilaru V, Marigorta UM, Mo A, Okou DT (2019). Blood-derived DNA methylation signatures of Crohn's disease and severity of intestinal inflammation. Gastroenterology.

[63] Shah SC, Khalili H, Gower-Rousseau C, Olen O, Benchimol EI, Lynge E (2018). Sex-based differences in incidence of inflammatory bowel diseases—pooled analysis of population-based studies from western countries. Gastroenterology.

[64] Swendsen C. Hypogonadism in Male Patients with Inflammatory Bowel Disease: 1540. Off J Am Coll Gastroenterol | ACG. 2012;107.

[65] Nasser M, Haider A, Saad F, Kurtz W, Doros G, Fijak M (2015). Testosterone therapy in men with Crohn's disease improves the clinical course of the disease: data from long-term observational registry study. Horm Mol Biol Clin Invest.

[66] Houseman EA, Molitor J, Marsit CJ (2014). Reference-free cell mixture adjustments in analysis of DNA methylation data. Bioinformatics.

[67] Chakravarthy A, Furness A, Joshi K, Ghorani E, Ford K, Ward MJ (2018). Pan-cancer deconvolution of tumour composition using DNA methylation. Nat Commun.

[68] Salas LA, Koestler DC, Butler RA, Hansen HM, Wiencke JK, Kelsey KT (2018). An optimized library for reference-based deconvolution of whole-blood biospecimens assayed using the Illumina HumanMethylationEPIC BeadArray. Genome Biol.

[69] Salas LA, Zhang Z, Koestler DC, Butler RA, Hansen HM, Molinaro AM, et al. Enhanced cell deconvolution of peripheral blood using DNA methylation for high-resolution immune profiling. bioRxiv. 2021. 10.1038/s41467-021-27864-7 PMC8828780 35140201

[70] Horvath S (2013). DNA methylation age of human tissues and cell types. Genome Biol.

[71] Marioni RE, Shah S, McRae AF, Chen BH, Colicino E, Harris SE (2015). DNA methylation age of blood predicts all-cause mortality in later life. Genome Biol.

[72] Cheung AS, Lim HY, Cook T, Zwickl S, Ginger A, Chiang C (2021). Approach to interpreting common laboratory pathology tests in transgender individuals. J Clin Endocrinol Metab.

[73] Aryee MJ, Jaffe AE, Corrada-Bravo H, Ladd-Acosta C, Feinberg AP, Hansen KD (2014). Minfi: a flexible and comprehensive Bioconductor package for the analysis of Infinium DNA methylation microarrays. Bioinformatics.

[74] Phipson B, Maksimovic J, Oshlack A (2016). missMethyl: an R package for analyzing data from Illumina's HumanMethylation450 platform. Bioinformatics.

[75] Maksimovic J, Gordon L, Oshlack A (2012). SWAN: subset-quantile within array normalization for illumina infinium HumanMethylation450 BeadChips. Genome Biol.

[76] Fortin JP, Labbe A, Lemire M, Zanke BW, Hudson TJ, Fertig EJ (2014). Functional normalization of 450k methylation array data improves replication in large cancer studies. Genome Biol.

[77] Pidsley R, Zotenko E, Peters TJ, Lawrence MG, Risbridger GP, Molloy P (2016). Critical evaluation of the Illumina MethylationEPIC BeadChip microarray for whole-genome DNA methylation profiling. Genome Biol.

[78] Ritchie ME, Phipson B, Wu D, Hu Y, Law CW, Shi W (2015). limma powers differential expression analyses for RNA-sequencing and microarray studies. Nucleic Acids Res.

[79] Du P, Zhang X, Huang CC, Jafari N, Kibbe WA, Hou L (2010). Comparison of Beta-value and M-value methods for quantifying methylation levels by microarray analysis. BMC Bioinformatics.

[80] Howe EA, Sinha R, Schlauch D, Quackenbush J (2011). RNA-Seq analysis in MeV. Bioinformatics.

[81] Novakovic B, Habibi E, Wang SY, Arts RJW, Davar R, Megchelenbrink W, et al. beta-Glucan reverses the epigenetic state of LPS-induced immunological tolerance. Cell. 2016;167(5):1354–68 e14. 10.1016/j.cell.2016.09.034 PMC5927328 27863248

[82] McLean CY, Bristor D, Hiller M, Clarke SL, Schaar BT, Lowe CB (2010). GREAT improves functional interpretation of cis-regulatory regions. Nat Biotechnol.

[83] Peters TJ, Buckley MJ, Statham AL, Pidsley R, Samaras K, R VL, et al. De novo identification of differentially methylated regions in the human genome. Epigenetics Chromatin. 2015;8:6. 10.1186/1756-8935-8-6 PMC4429355 25972926

[84] Quinlan AR, Hall IM (2010). BEDTools: a flexible suite of utilities for comparing genomic features. Bioinformatics.

[85] Cotton AM, Price EM, Jones MJ, Balaton BP, Kobor MS, Brown CJ (2015). Landscape of DNA methylation on the X chromosome reflects CpG density, functional chromatin state and X-chromosome inactivation. Hum Mol Genet.

[86] Inoshita M, Numata S, Tajima A, Kinoshita M, Umehara H, Yamamori H (2015). Sex differences of leukocytes DNA methylation adjusted for estimated cellular proportions. Biol Sex Differ.

[87] Heinz S, Benner C, Spann N, Bertolino E, Lin YC, Laslo P (2010). Simple combinations of lineage-determining transcription factors prime cis-regulatory elements required for macrophage and B cell identities. Mol Cell.

