# Supplementary material for: Gender-affirming hormone therapy induces specific DNA methylation changes in blood
Source: Clin Epigenetics. 2022 Feb 17;14:24. doi: 10.1186/s13148-022-01236-4 (PMC8851870; doi:10.1186/s13148-022-01236-4)
Supplement: Supplementary file 10 — Additional file 10: Fig. S1. Summary of covariates used in the EWAS analysis. A. PCA loadings in B. Heatmap highlighting the contribution of different variables to each principal component. Technical variation, such as location of sample in the EPIC array, was the biggest contributor to variation, followed by cell composition. The top number in each box within the heatmap is the correlation between principal component and data traits and underneath in brackets are the p values. Covariates with a high correlation and low p value are highlighted in red for positive correlations and blue for negative correlations. Fig. S2. A. Bar plot showing change in DNA methylation (Δβ) relative to baseline for top DMPs in the baseline vs 6 months or baseline vs 12 months comparison for transgender women. B. Bar plot showing change in DNA methylation (Δβ) or top DMPs in transgender men. C-F. Line plot showing DNA methylation changes per donor (different colour per donor) over time for selected DMPs. The mean DNA methylation level is shown as a bar plot. The X-axis refers to the GAHT group and time: TW = Transgender women, TM = Transgender men. Fig. S3. DNA methylation signature separates baseline from 6-month and 12-month GAHT samples. A. Scatter plot of change in DNA methylation relative to baseline for DMPs that are significant at both 6m and 12m following feminizing GAHT. There is a positive correlation between 6m-baseline Δβ (x axis) and 12m-baseline Δβ (y axis). B. Scatter plot of change in DNA methylation relative to baseline for DMPs that are significant at both 6m and 12m following masculinizing GAHT. There is a positive correlation between 6m-baseline Δβ (x axis) and 12m-baseline Δβ (y axis). C. Direction-of-change Venn diagram depicting overlap of 6m and 12m gain or loss of methylation DMPs in transgender women (TW). D. Direction-of-change Venn diagram depicting overlap of 6m and 12m gain or loss of methylation DMPs in transgender men (TM). In C. and D., we observe that ov [file 13148_2022_1236_MOESM10_ESM.pdf]

A

PCA plot of 1st 10 principal components

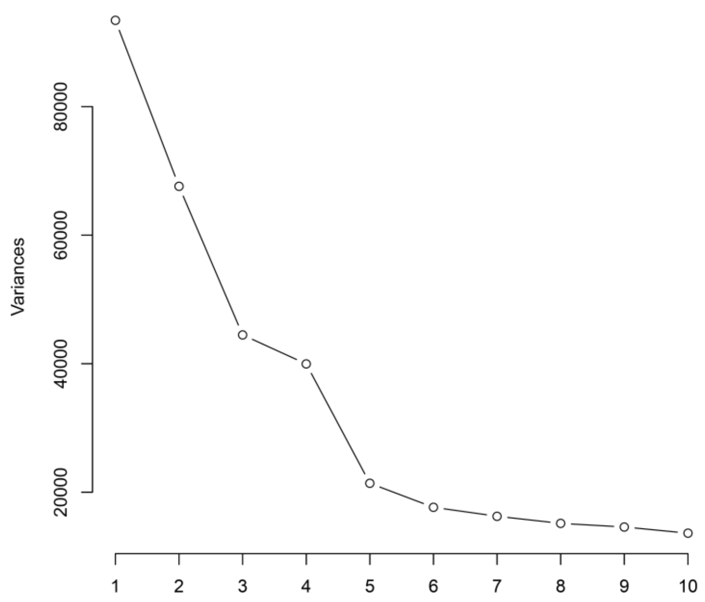

B

PCA – Confounders relationships
'correlation (p-value)'

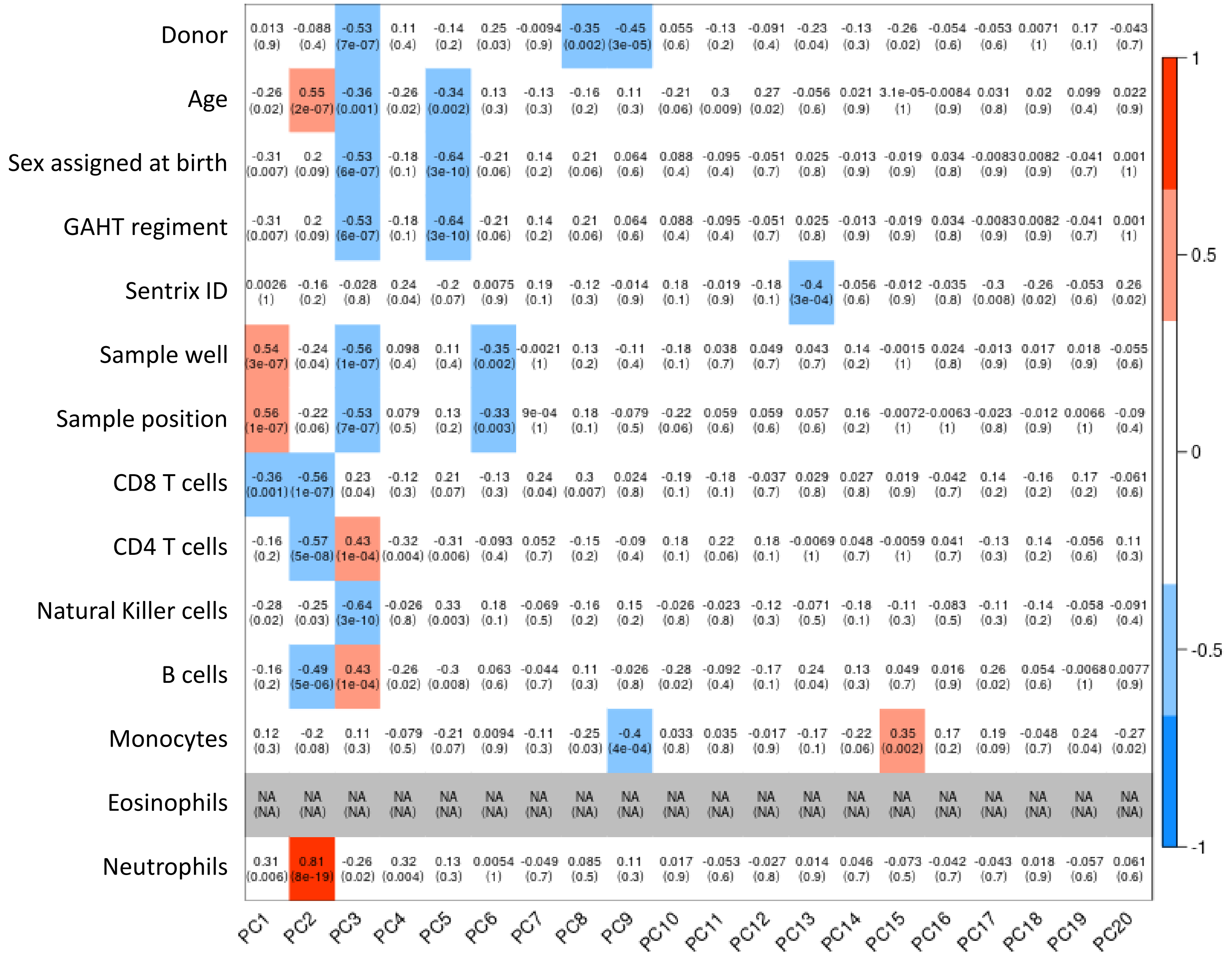

Supplemental Figure 1

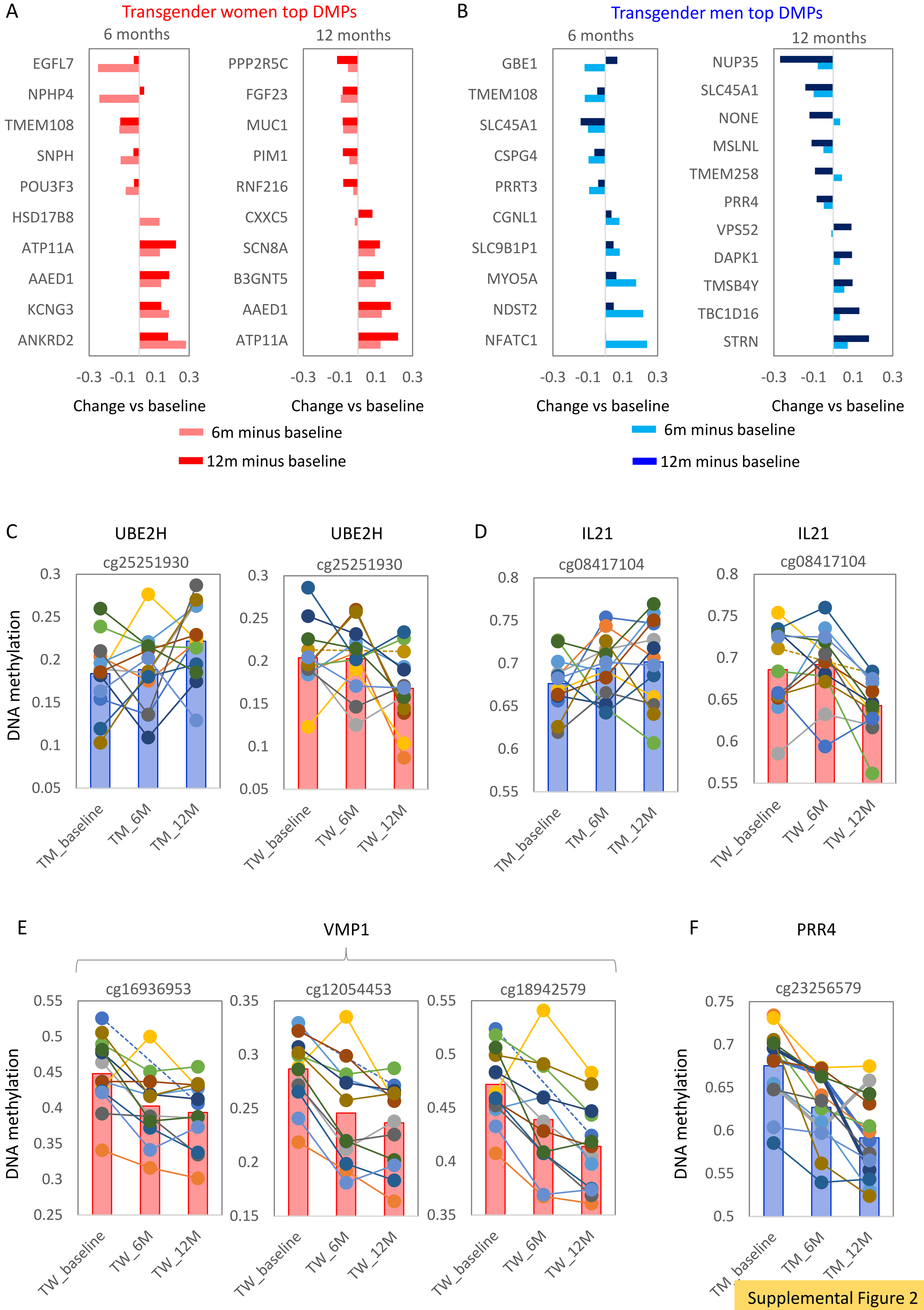

Supplemental Figure 2

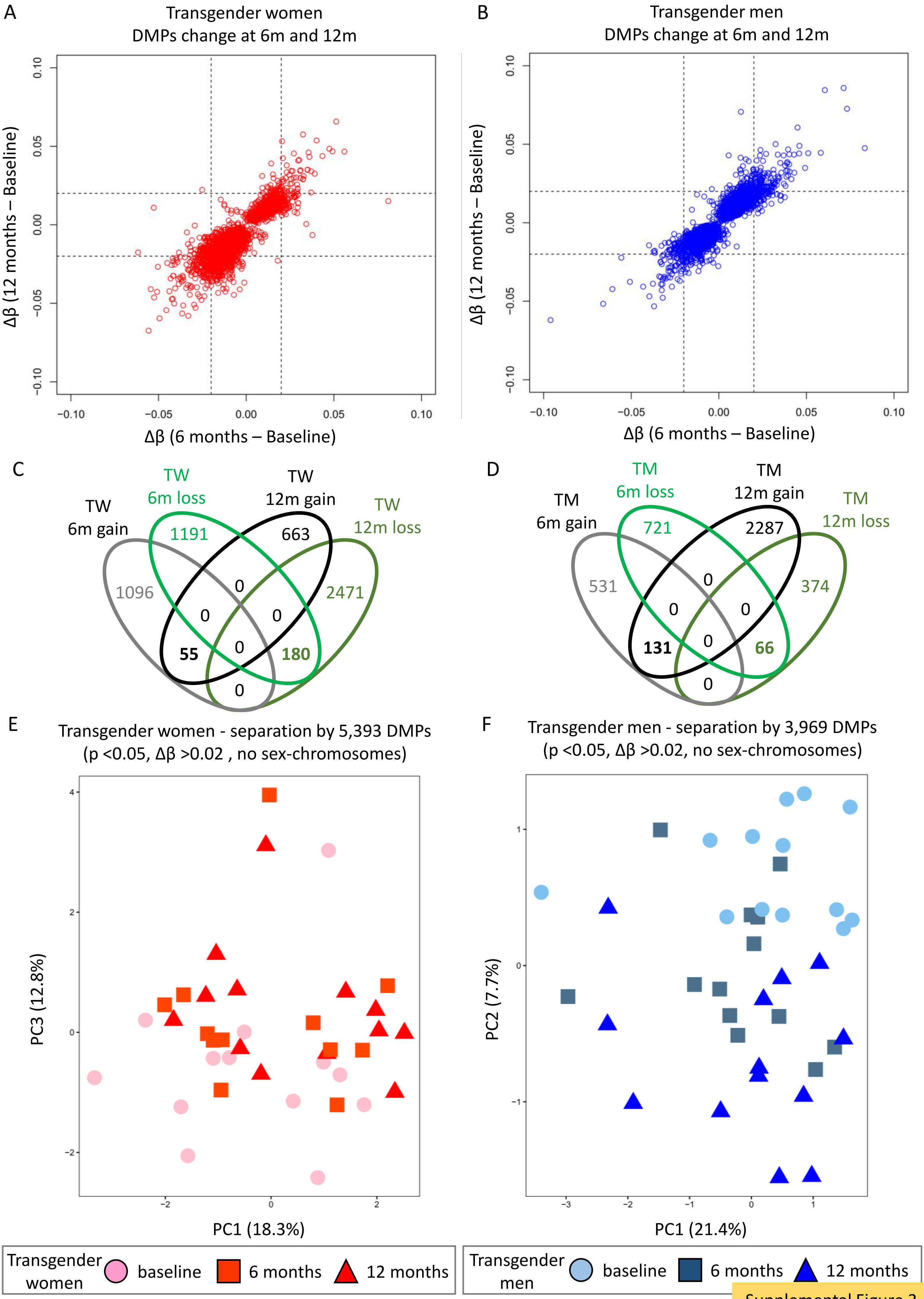

Supplemental Figure 3

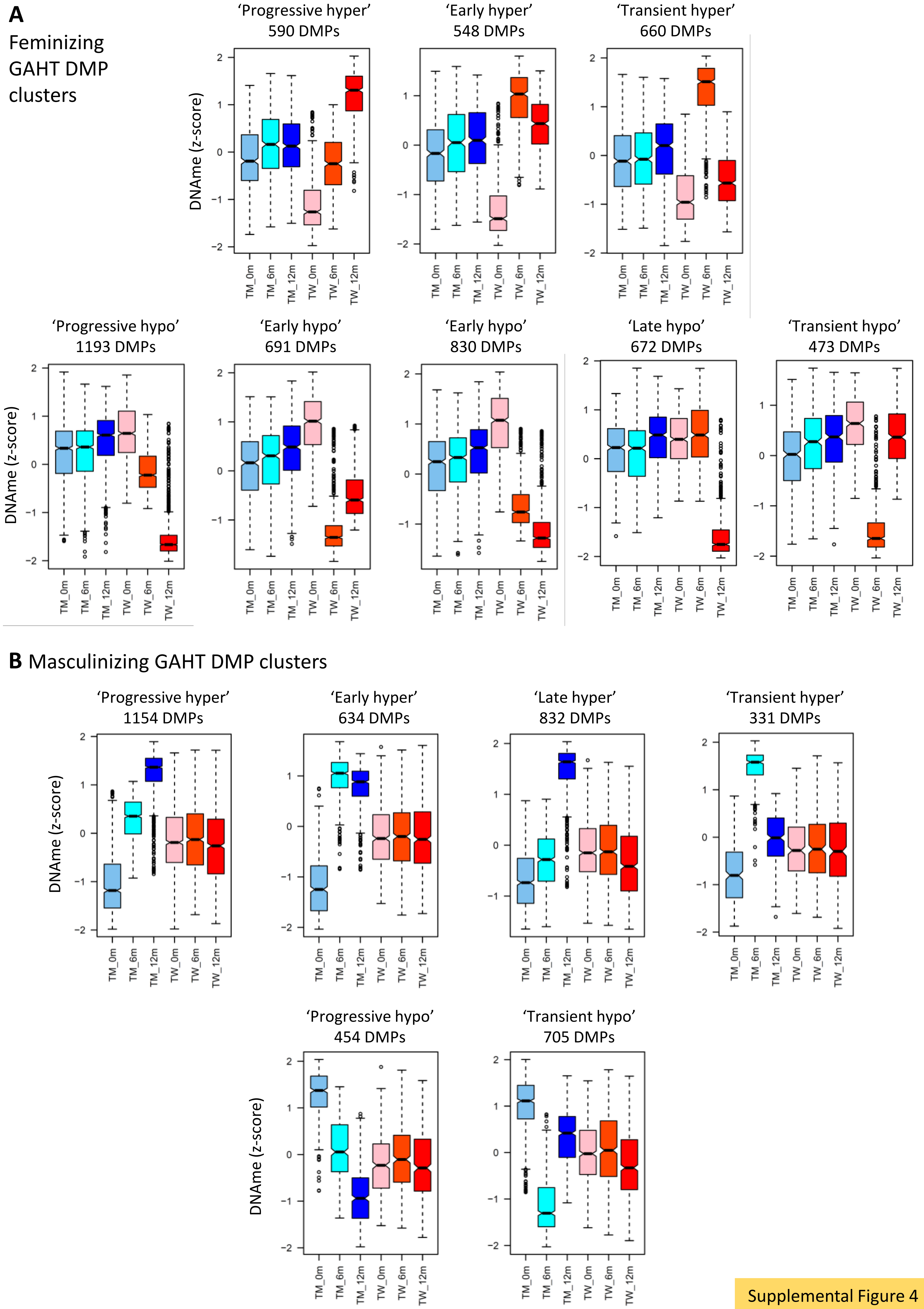

Supplemental Figure 4

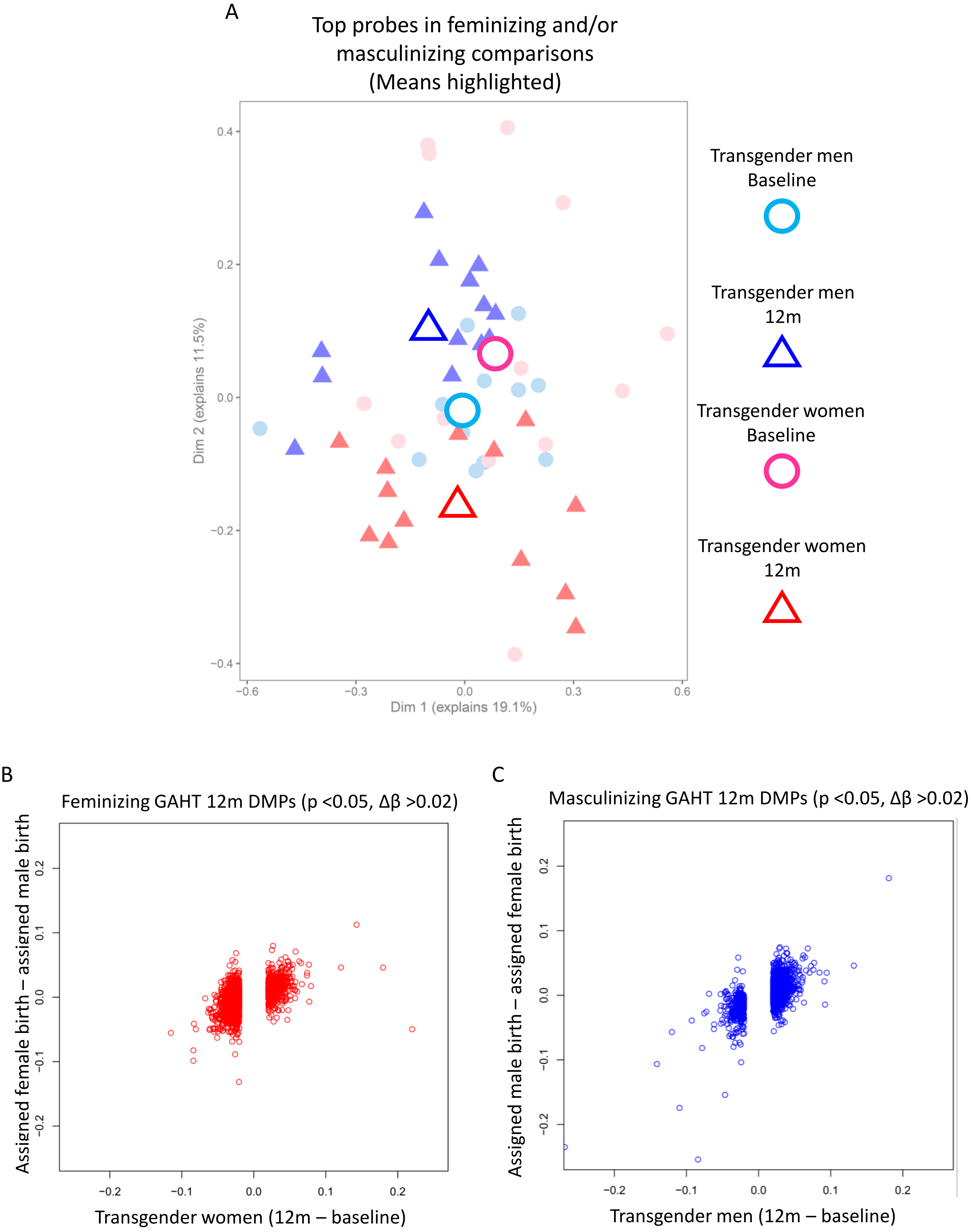

Supplemental Figure 5

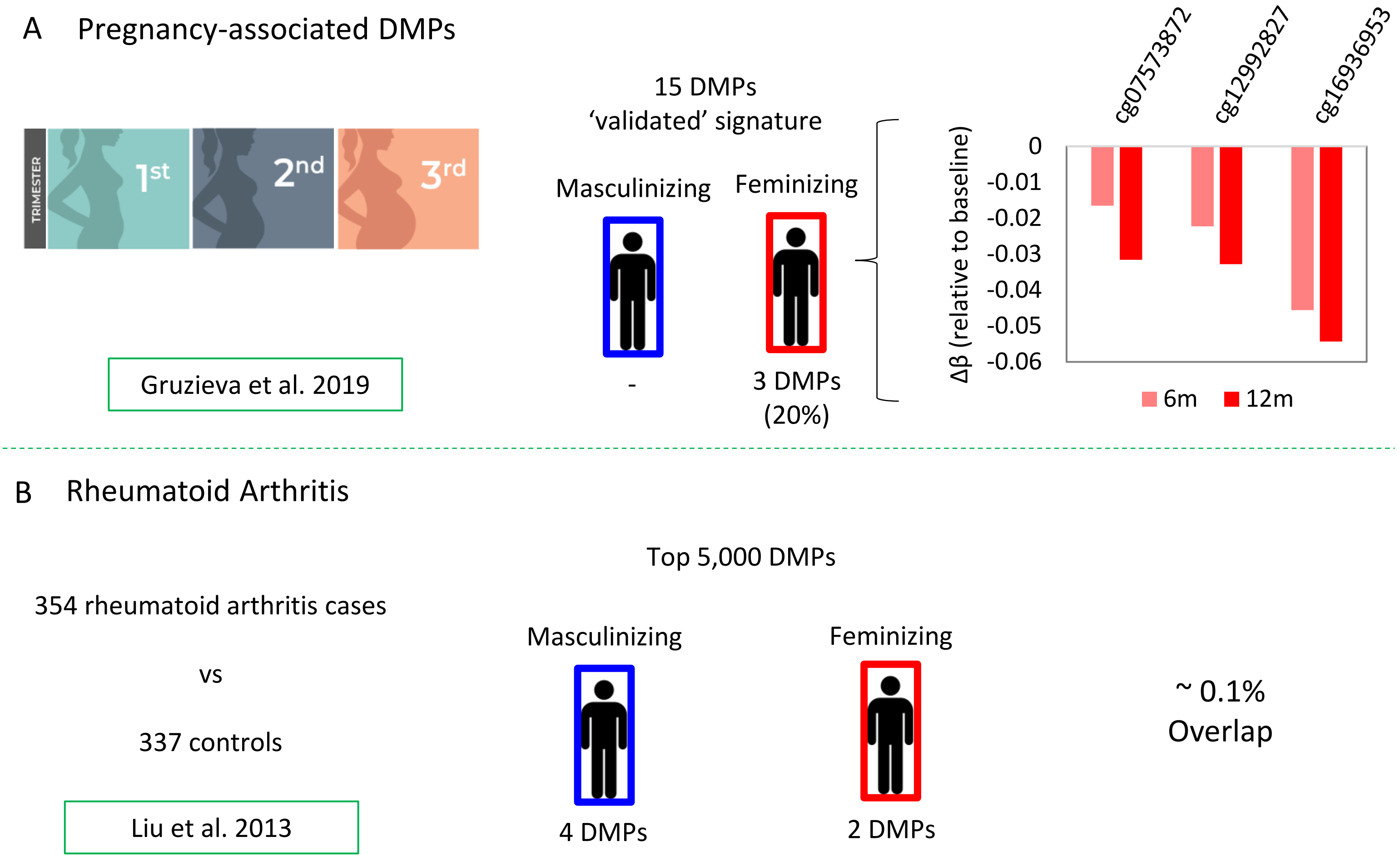

Supplemental Figure 6

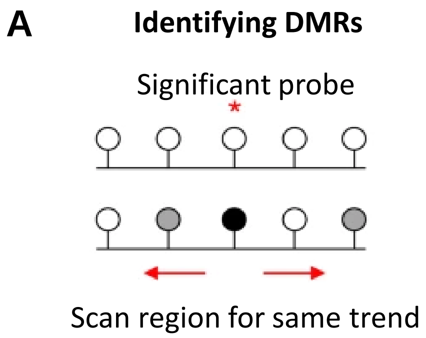

B

Feminizing GAHT (6 months v baseline):
6 DMRs: near *TAS1R2, ZFP36L1, RTL1, MED31, VMP1, SLC35D3*

Feminizing GAHT(12 months v baseline):
3 DMRs: near *CAT, LANCL3, HUS1*

Masculinizing GAHT (6 months v baseline):
0 DMRs:

Masculinizing GAHT (12 months v baseline):
3 DMRs: near *PRR4, PHF19, MMP17*

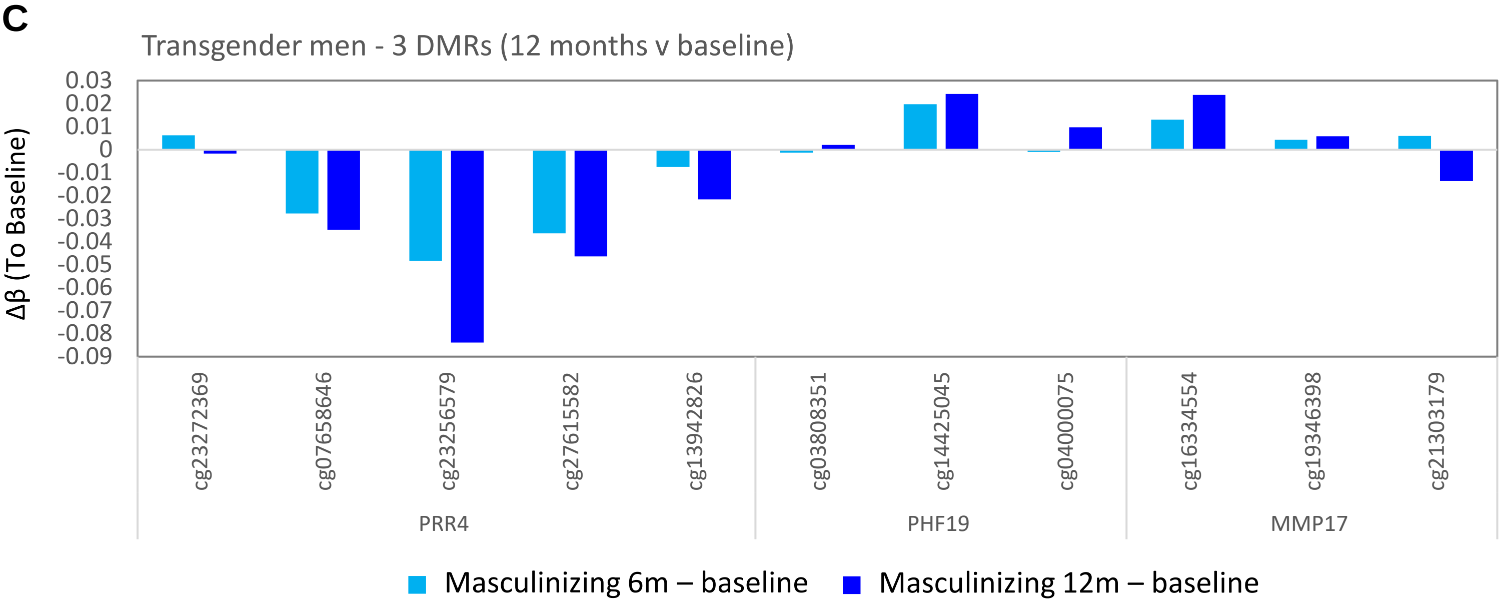

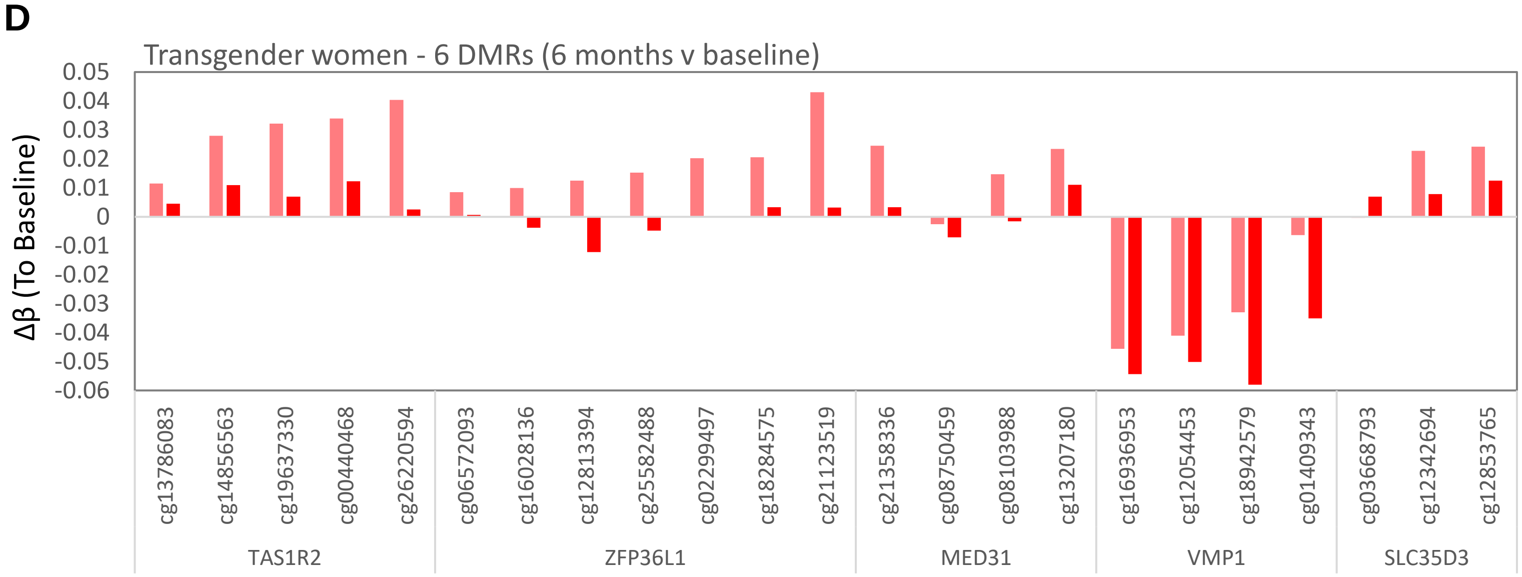

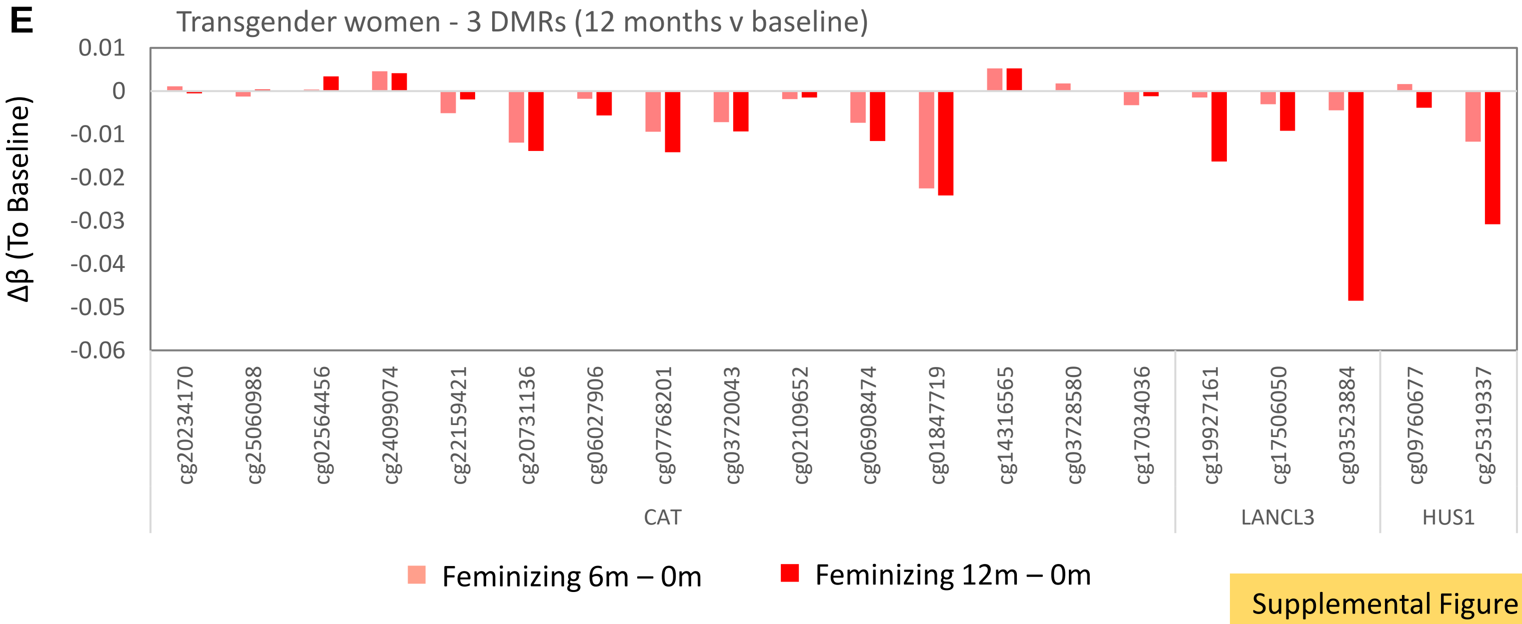

Supplemental Figure 7

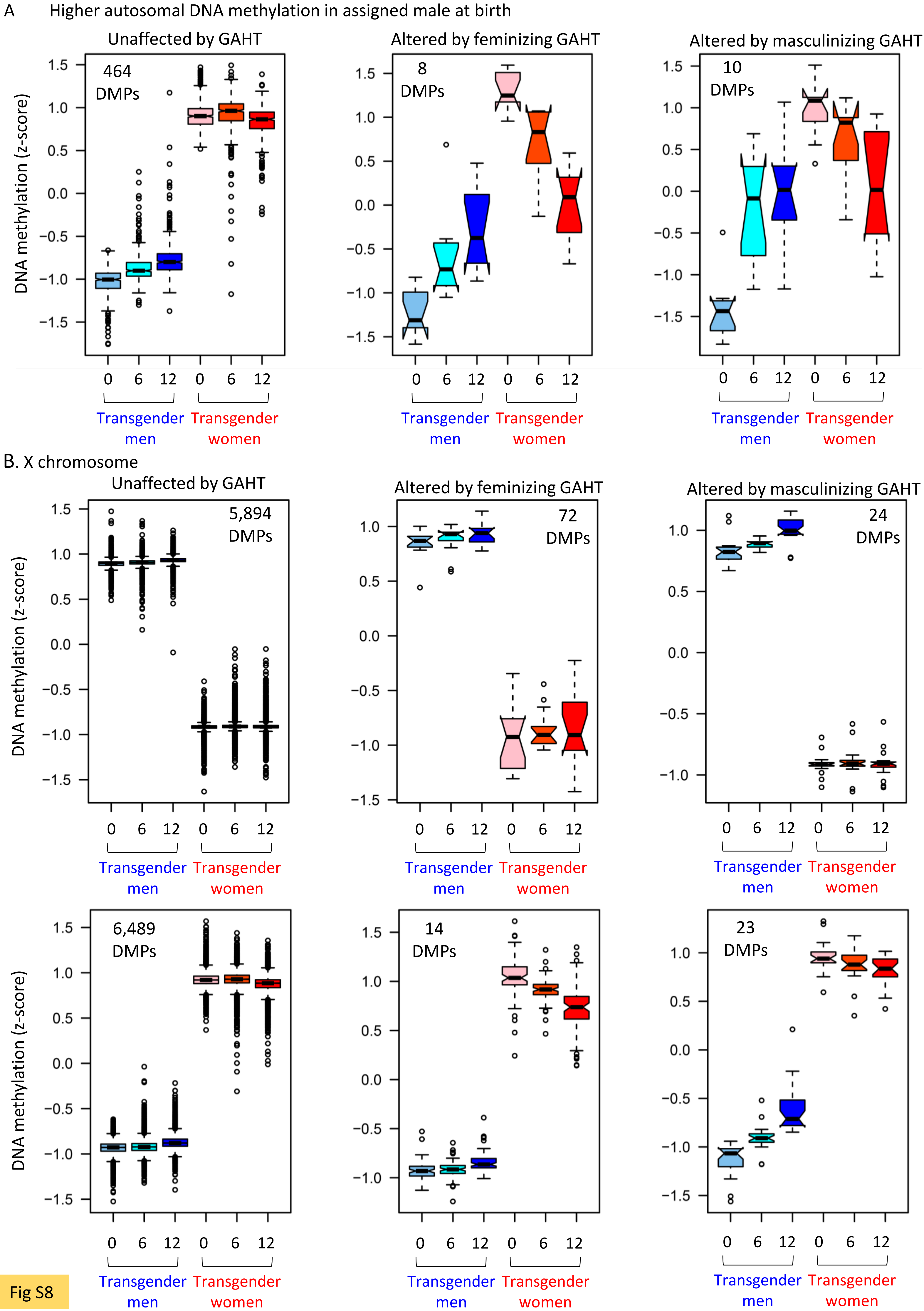
A Higher autosomal DNA methylation in assigned male at birth
Unaffected by GAHT
464 DMPs
Altered by feminizing GAHT
8 DMPs
Altered by masculinizing GAHT
10 DMPs
DNA methylation (z-score)
0 6 12 0 6 12
Transgender men
Transgender women
B. X chromosome
Unaffected by GAHT
5,894 DMPs
Altered by feminizing GAHT
72 DMPs
Altered by masculinizing GAHT
24 DMPs
6,489 DMPs
14 DMPs
23 DMPs

Fig S8

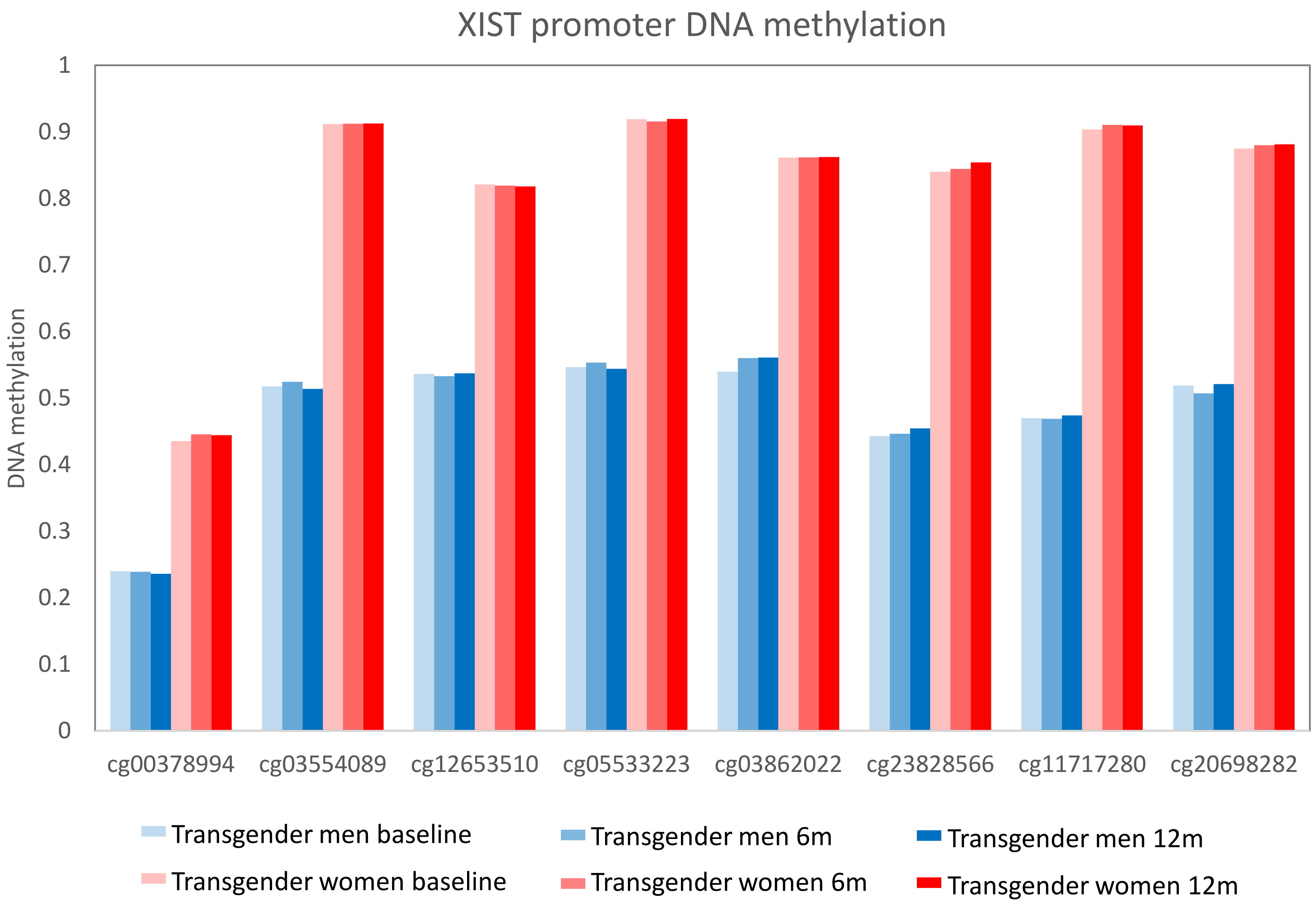
XIST promoter DNA methylation
DNA methylation
1
0.9
0.8
0.7
0.6
0.5
0.4
0.3
0.2
0.1
0
cg00378994
cg03554089
cg12653510
cg05533223
cg03862022
cg23828566
cg11717280
cg20698282
Transgender men baseline
Transgender men 6m
Transgender men 12m
Transgender women baseline
Transgender women 6m
Transgender women 12m

Figure S9

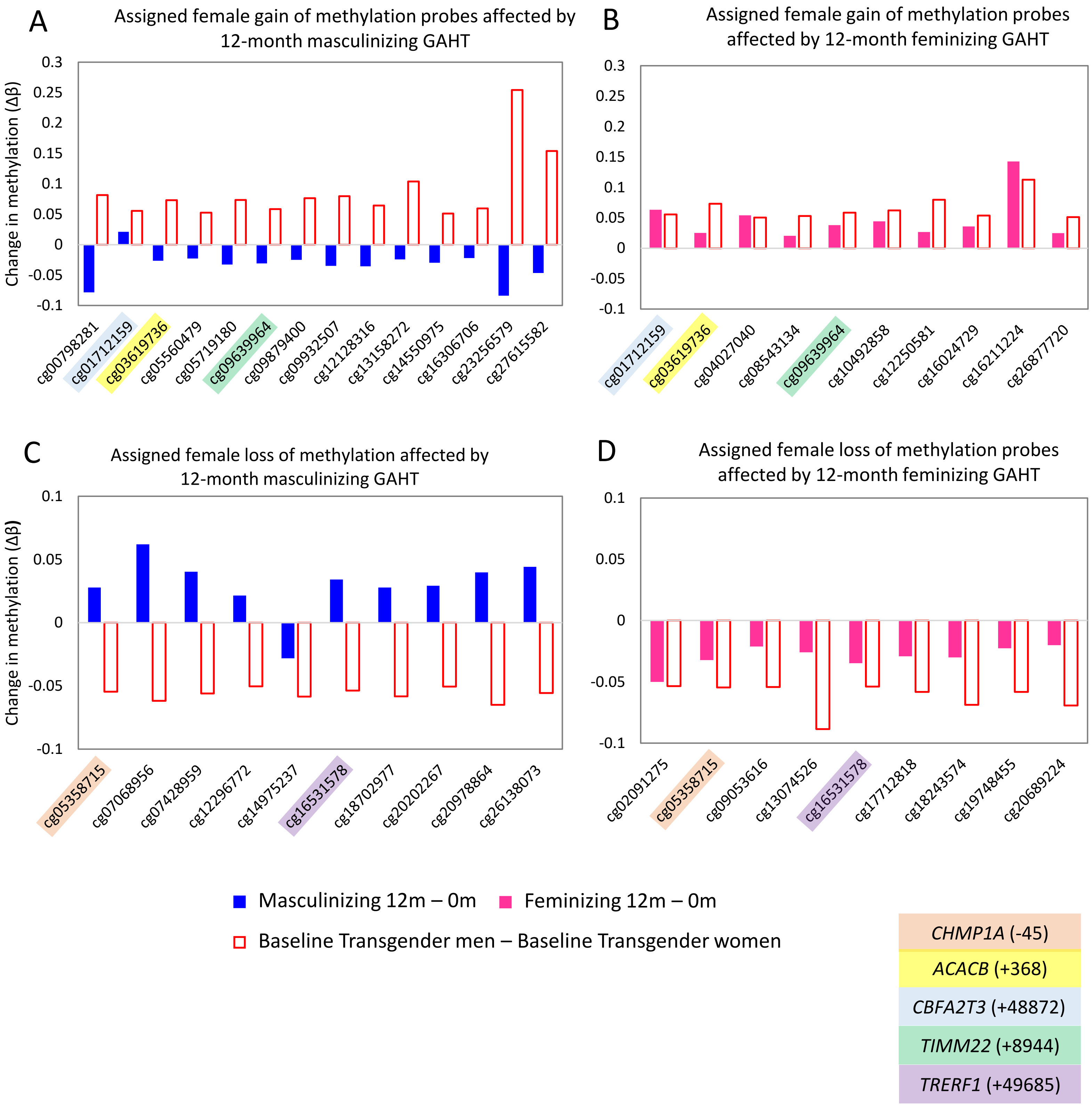
A
Assigned female gain of methylation probes affected by 12-month masculinizing GAHT
B
Assigned female gain of methylation probes affected by 12-month feminizing GAHT
C
Assigned female loss of methylation affected by 12-month masculinizing GAHT
D
Assigned female loss of methylation probes affected by 12-month feminizing GAHT
Change in methylation (Δβ)
cg00798281
cg01712159
cg03619736
cg05560479
cg05719180
cg09639964
cg09879400
cg09932507
cg12128316
cg13158272
cg14550975
cg16306706
cg23256579
cg27615582
cg04027040
cg08543134
cg10492858
cg12250581
cg16024729
cg16211224
cg26877720
cg05358715
cg07068956
cg07428959
cg12296772
cg14975237
cg16531578
cg18702977
cg20202267
cg20978864
cg26138073
cg02091275
cg09053616
cg13074526
cg17712818
cg18243574
cg19748455
cg20689224
Masculinizing 12m – 0m
Feminizing 12m – 0m
Baseline Transgender men – Baseline Transgender women
CHMP1A (-45)
ACACB (+368)
CBFA2T3 (+48872)
TIMM22 (+8944)
TRERF1 (+49685)

Figure S10
